# Structure formation during translocon-unassisted co-translational membrane protein folding

**DOI:** 10.1038/s41598-017-08522-9

**Published:** 2017-08-14

**Authors:** Nicola J. Harris, Eamonn Reading, Kenichi Ataka, Lucjan Grzegorzewski, Kalypso Charalambous, Xia Liu, Ramona Schlesinger, Joachim Heberle, Paula J. Booth

**Affiliations:** 1 Department of Chemistry, Britannia House, 7 Trinity Street, King’s College London, London, UK; 2 Department of Physics, Freie Universität Berlin, Arnimallee 14, 14195 Dahlem, Germany; 3 School of Biochemistry, Medical Sciences, University Walk, University of Bristol, Bristol, UK

## Abstract

Correctly folded membrane proteins underlie a plethora of cellular processes, but little is known about how they fold. Knowledge of folding mechanisms centres on reversible folding of chemically denatured membrane proteins. However, this cannot replicate the unidirectional elongation of the protein chain during co-translational folding in the cell, where insertion is assisted by translocase apparatus. We show that a lipid membrane (devoid of translocase components) is sufficient for successful co-translational folding of two bacterial α-helical membrane proteins, DsbB and GlpG. Folding is spontaneous, thermodynamically driven, and the yield depends on lipid composition. Time-resolving structure formation during co-translational folding revealed different secondary and tertiary structure folding pathways for GlpG and DsbB that correlated with membrane interfacial and biological transmembrane amino acid hydrophobicity scales. Attempts to refold DsbB and GlpG from chemically denatured states into lipid membranes resulted in extensive aggregation. Co-translational insertion and folding is thus spontaneous and minimises aggregation whilst maximising correct folding.

## Introduction

The folding of proteins from an amino acid sequence to their correct functional state is a fundamental process that is essential to the biological activity of living systems. As such, folding has garnered considerable attention, but this has primarily focussed on water-soluble proteins with considerably less information being available for the hydrophobic proteins that reside within cell membranes.

Co-translational folding is a dominant pathway in cells, particularly for the vast class of transmembrane (TM) α-helical proteins. Insertion into, and folding within, the membrane occurs during the linear elongation of the polypeptide sequence from the N to C terminus. However, the current mechanistic understanding of folding for both soluble and membrane proteins stems largely from refolding chemically denatured states. α-helical membrane proteins are difficult to denature, thus the denatured states of these proteins used in folding studies usually have significant residual secondary structure, and key structure formation events such as helix formation are missed^1, 2^. It is unclear how the results from these studies on denatured, isolated proteins relate to co-translational folding and insertion.

Co-translational membrane insertion of α-helical proteins occurs primarily via the translocon Sec system in eukaryotes and prokaryotes, for example SecYEG in *Escherichia coli* (*E. coli*)^3, 4^, or via other insertases, such as YidC^5^. In the current model for α-helical membrane protein insertion α-helical TM segments emerge from the ribosome and enter the pore of the translocon, partitioning into the membrane via a lateral gate. Recently an alternate model was proposed^6^ based on thermodynamics, in which some segments of the emerging nascent chain can associate more favourably with the membrane interface, and not with the translocon. TM helices can partition into the membrane, driven by the transfer free energy between aqueous and lipidic phases. This is supported by the recent finding that spontaneous TM insertion thermodynamically mimics translocon-guided insertion^7^. This alternative model raises the question as to whether spontaneous insertion and folding of the whole nascent chain can occur co-translationally, without the presence of any translocon components.

Previous work^8–13^ has shown that the mechanical energy stored in a lipid membrane bilayer modulates membrane protein folding, stability and function. Thus, we propose that manipulation of bilayer properties via lipid composition changes will facilitate successful insertion of nascent membrane proteins without the need for the translocon.

Cell-free systems provide a promising avenue to probe co-translational folding. These use cellular extracts or reconstituted translation systems to produce membrane proteins in the presence of supplied membranes or membrane mimetic environments, such as detergents, nanodiscs, bicelles, liposomes, and membrane extracts. Many studies have revealed that a variety of membrane proteins can be synthesised into a lipid membrane or detergent environment in the absence of a translocon, albeit sometimes with cellular extracts/components present^14–22^.

Building upon the success of cell-free methods, we use an efficient experimental system entirely devoid of translocon components for mechanistic studies of co-translational folding of α-helical membrane proteins. This allows the study of folding during the natural linear elongation of the nascent chain, which cannot be mimicked in denaturant studies. We address several key questions: is insertion and folding directly into the lipid membrane, without any translocon apparatus, co-translational? Does the lipid composition, and thus associated headgroup charge and bilayer mechanical properties, modulate membrane insertion yield? Do membrane proteins fold through different co-translational pathways, and if so, is it linked to their amino acid sequence? Can the thermodynamic free energy alone (translocase unassisted) produce folded and functional membrane proteins akin to those produced *in vivo*? Is co-translational folding advantageous in avoiding aggregation that hinders folding from chemically denatured states?

**Figure 1:**
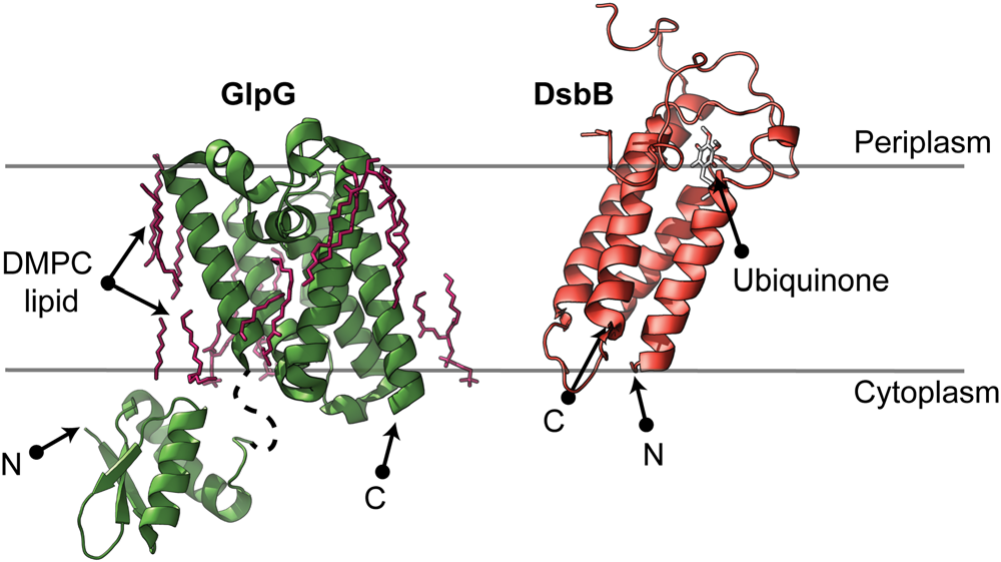
GlpG and DsbB protein structures. Crystal structures of GlpG (membrane domain PDB 2XTV; cytoplasmic N-terminal domain PDB 2LEP), with 6 TM helices and a soluble cytoplasmic domain (green, left), and DsbB (PDB 2LTQ) with 4 TM helices and a large periplasmic loop (red, right). The membrane boundaries (grey lines) and spatial arrangements for both membrane proteins were calculated by the orientations of proteins in membranes database (OPM)^52^. Both the N- and C-terminal ends of the crystal structure are labelled as N and C respectively.

Here, we compare two bacterial α-helical membrane proteins, GlpG and DsbB (Fig. 1 and Fig. S1). The *E. coli* rhomboid protease GlpG is a six TM integral membrane protein with an intracellular domain at its N-terminus. The bacterial protein DsbB from *E. coli* is a four TM integral membrane protein which is a component of the disulphide bond formation pathway in the periplasm. High resolution structures^23, 24^ and activity assays^23, 25^ are known for both GlpG and DsbB, together with stability, mutant and sodium dodecyl sulphate (SDS) denaturation studies^26–31^, making them ideal candidates to study membrane-dependent co-translational folding and insertion. We employ a recently devised vibrational spectroscopic method (Surface-Enhanced InfraRed Absorption Spectroscopy (SEIRAS)^32^) to probe whether structure formation occurs co-translationally. Infrared (IR) spectroscopy can resolve protein structure formation through changes in intrinsic protein bond vibrations without any perturbing protein labelling. Moreover, time-resolved IR gives information on the folding dynamics with high spatial sensitivity, which is especially useful for kinetic measurements of membrane protein folding.

Our comparison of GlpG and DsbB gives unprecedented structural insight into membrane insertion and co-translational folding of membrane proteins. This progresses earlier denaturant studies on GlpG^1, 27^ and DsbB^26, 28, 30^, and on membrane proteins in general, which use chemically denatured states containing some or all α-helical secondary structure^1^.

## Results

### Cell-free synthesis in the presence of a lipid membrane can produce spontaneously inserted GlpG and DsbB

**Figure 2:**
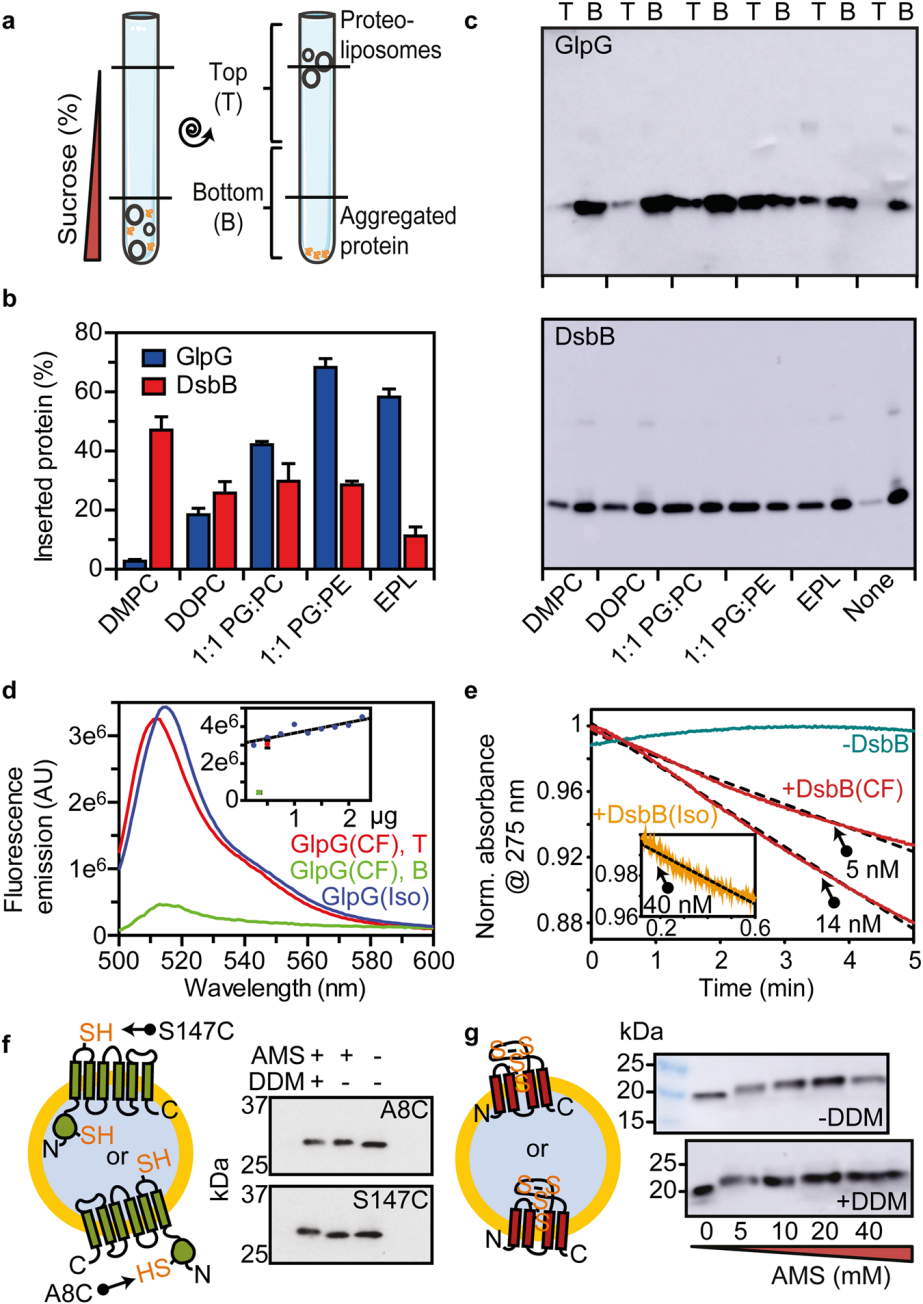
GlpG and DsbB cell-free synthesis. (**a**) Sucrose flotation assay to separate proteoliposomes (top, T) from non-inserted and aggregated protein (bottom, B). (**b**) The amount of inserted protein in the floated proteoliposomes was quantified via the [^14^C]-Leucine or [^35^S]-Methionine incorporated during protein synthesis, and expressed as a percentage of the total protein synthesised. Error bars are the SEM from at least three repeats. (**c**) Western blot analysis of the top and bottom sucrose gradient fractions of cell-free produced GlpG and DsbB. (**d**) Fluorescence spectra of GlpG protease activity of a soluble BODIPY-tagged casein. Cell-free (CF, top (red) and bottom (green)) or *in vivo* expressed and isolated (Iso, blue) GlpG were in DOPE:DOPG (1:1 mol ratio) liposomes. Inset: the fluorescence band maxima of GlpG protease activity versus GlpG concentration. (**e**) DsbB redox functional assay with DsbA and ubiquinone; reduction of ubiquinone results in a strong decrease in absorbance at 275 nm. Cell-free (CF, red) or *in vivo* expressed and isolated (Iso, orange) DsbB were in DMPC liposomes; a control sample without DsbB (green) is shown. Least-squared fitted linear fits (dashed black lines) calculating the initial rate of quinone reduction are shown. Possible topology of GlpG (**f**) and DsbB (**g**) after cell-free synthesis and insertion into liposomes (gold membrane with blue liposome interior). GlpG mutants (A8C and S147C) were used as AMS labelling sites to interrogate topology. DsbB contains two disulphide bonds within its periplasmic loops which, if exposed on the outside of the liposome, become available for AMS labelling upon TCEP reduction.

GlpG and DsbB were produced using a transcription-translation linked cell-free system^14^ in the presence of liposomes. Once the cell-free reaction was complete, non-inserted and aggregated protein were removed by mixing with sucrose and urea and floating on a sucrose gradient (Fig. 2a)^33^. Empty liposomes and proteoliposomes float to the buffer/sucrose interface at the top of the sucrose gradient. The floated proteoliposomes contained GlpG or DsbB, consistent with spontaneous insertion of these proteins. The bottom of the sucrose gradient contained aggregated and non-inserted protein, but negligible liposomes (Fig. S2). The ribosomes remain at the bottom of the sucrose gradient, as they do not associate with membranes unless they contain translating or translation stalled nascent chains^34^. Cell-free synthesis of GlpG and DsbB was also performed in the presence of liposomes of different lipid compositions. Five lipid compositions were measured: 100% 1,2-dimyristoyl-*sn*-glycero-3-phosphocholine (DMPC), 100% 1,2-dioleoyl-*sn*-glycero-3-phosphocholine (DOPC), 100% *E. coli* polar lipid extract (EPL) and 1:1 mol ratios of 1,2-dioleoyl-*sn*-glycero-3-phosphoglycerol (DOPG):DOPC and DOPG: 1,2-dioleoyl-*sn*-glycero-3-phosphoethanolamine (DOPE).

The total amount of protein expressed by the cell-free system was quantified via the amount of [^14^C]-Leucine or [^35^S]-Methionine incorporated during synthesis. Around 120–170 µg.ml^−1^ of GlpG was produced in total (around 3.5–5 µM), and around 50–100 µg.ml^−1^ DsbB (around 1.5–4.5 µM) in a 25 µl cell-free reaction. The degree of protein insertion into liposomes was quantified by comparing the amount of protein present in the proteoliposomes in the top layer of the sucrose gradient with the total amount synthesised (Fig. 2b). Thus, insertion yields are quoted as the percentage of total protein synthesised. Western blot analysis was used to qualitatively assess the protein purity, quality and aggregation of GlpG and DsbB following cell-free synthesis and insertion (Fig. 2c). In all lipid compositions tested the inserted fraction of both proteins contained negligible oligomeric aggregated species.

The lipid composition influenced the yield of spontaneously inserted GlpG and DsbB, with a greater effect for GlpG, ranging from 3% yield in DMPC to 68% in a 1:1 mol ratio DOPG:DOPE. DsbB yields ranged from 11–47%. The GlpG insertion yield increased from that in DMPC when unsaturation is introduced in the lipid chains (i.e. for DOPC), and when PG or PE headgroups are introduced. In contrast DsbB inserts best into DMPC bilayers, with lower insertion yields observed when PG or PE headgroups are introduced. The biggest difference in spontaneous insertion between the two proteins occurs in DMPC; only 3% of GlpG spontaneously inserts into DMPC liposomes compared to DsbB where 47% inserts. Alteration in lipid chain saturation, from saturated DMPC to unsaturated DOPC, decreased DsbB insertion (47% to 26%, respectively), while GlpG increased (3% to 18%, respectively).

Correct folding was assessed by functional assays of cell-free expressed and spontaneously inserted GlpG and DsbB. While GlpG is an intramembrane protease, GlpG can cleave the soluble protein casein from the fluorescent group BODIPY, providing a convenient substrate to assay GlpG activity^23^. This cleavage unquenches the BODIPY fluorescence, and fluorescent emission can be used as a measure of GlpG activity. This soluble substrate assay works for GlpG in detergent or lipid membranes, occurring at a slower rate in membranes^35^. The activity of cell-free produced GlpG proteoliposomes was compared to the activity of isolated GlpG reconstituted into liposomes. In line with previous studies^23, 31, 36^, an endpoint assay was used to measure activity, as the rate of protease activity is highly influenced by the surrounding bilayer^35^. We found the endpoint to be dependent on the protein concentration for reconstituted GlpG (Fig. 2d). Cell-free GlpG in 1:1 DOPG:DOPE liposomes had a similar end point as reconstituted GlpG (~3 × 10^6^ AU at the same protein concentration), both yielding the characteristic emission band of the cleaved fluorescent BODIPY group at around 520 nm.

Cell-free produced DsbB was probed for function using an assay which monitors the reduction of ubiquinone (UQ-1) during the redox reaction between DsbB and DsbA *in vitro* ^25^ (Table S1). Cell-free DsbB was found to be functional with a specific activity in DMPC liposomes (Fig. 2e, 236.8 ± 3.3 and 146.4 ± 0.4 nmol quinone per nmol of DsbB min^−1^) similar to that of recombinantly expressed and detergent solubilized DsbB (Fig. S2, 128.4 ± 0.6 nmol quinone per nmol of DsbB min^−1^), as well as that from isolated DsbB reconstituted into DMPC liposomes (Fig. 2e, 124.9 ± 2.0 nmol quinone per nmol of DsbB min^−1^) and cell membranes containing over-expressed DsbB^25^. This advocates that spontaneously inserted DsbB is folded and functional.

The topology of spontaneously inserted GlpG and DsbB was also investigated. Labelling of exposed cysteine residues with the non-membrane permeable label AMS confirmed that GlpG inserted into the liposomes with its soluble N-terminal domain on the outside of the liposome (Fig. 2f), consistent with its cellular topology. Interestingly, the AMS labelling experiments suggest that DsbB inserted with its periplasmic loops exposed on the outside of the liposome (Fig. 2g and Supplementary Discussion) – an opposite topology to that found for DsbB *in vivo* and for GlpG.

### Structure formation during cell-free membrane insertion and folding of GlpG and DsbB

SEIRAS was used to study the structure formation of GlpG and DsbB during their synthesis by the ribosome. SEIRAS uses gold surface enhancement to measure IR spectroscopy at a range of 10 nm from a gold surface giving 10–100 times the sensitivity of conventional IR absorption spectroscopy^37^.

In this comparative study, we used surface tethered DMPC nanodiscs, which have already proven successful in co-translational SEIRAS studies^32^. The nanodiscs fall within the 10 nm surface enhancement range, meaning that protein that is not directly associated with the lipids is undetected. In view of the lipid dependence of insertion, future work will develop the method for different lipid compositions and temperatures. It is of note, however, that lipids in nanodiscs have altered phase behaviour than in liposomes or bilayers. DMPC has a significantly broader gel to liquid crystalline phase transition temperature in nanodiscs (between 20–40 °C) than in liposomes^38^. This is due to the nanodiscs possessing both a much smaller lipid cooperative unit compared to liposomes and a significant amount of boundary lipid interacting with the protein. However, biological membranes contain high percentages of boundary lipids and proteins, therefore it has been argued that a nanodisc may represent a more native-like membrane environment than dilute membrane proteins in liposomes^38^. Both GlpG and DsbB were functional in DMPC nanodiscs after cell-free insertion and folding (Fig. S2).

**Figure 3:**
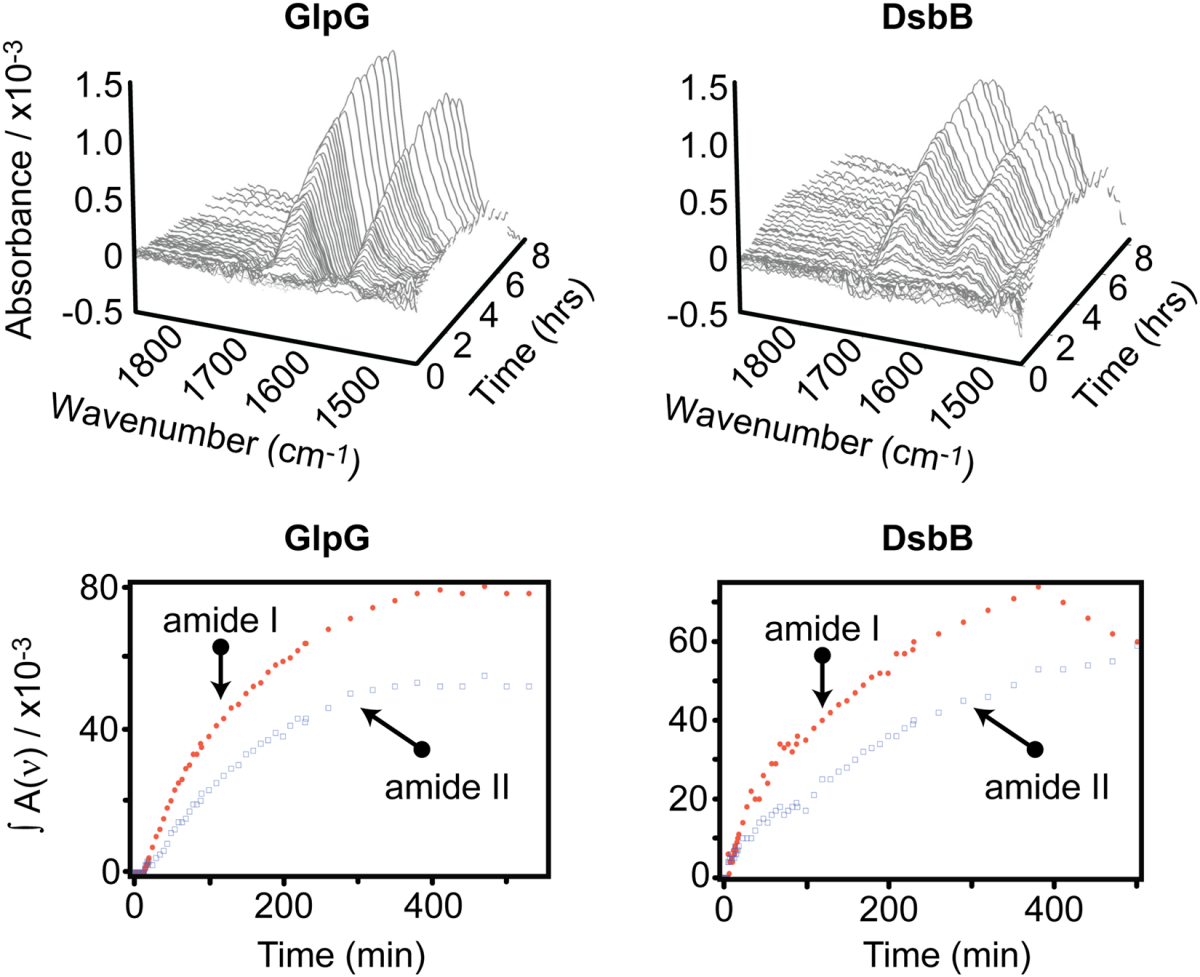
Time-resolved SEIRAS spectra of GlpG and DsbB. (Top row) Amide I and II peaks of GlpG (left) and DsbB (right) during cell-free synthesis and insertion into DMPC nanodiscs. (Bottom row) Time course of the amide I and amide II integrated peak areas.

**Figure 4:**
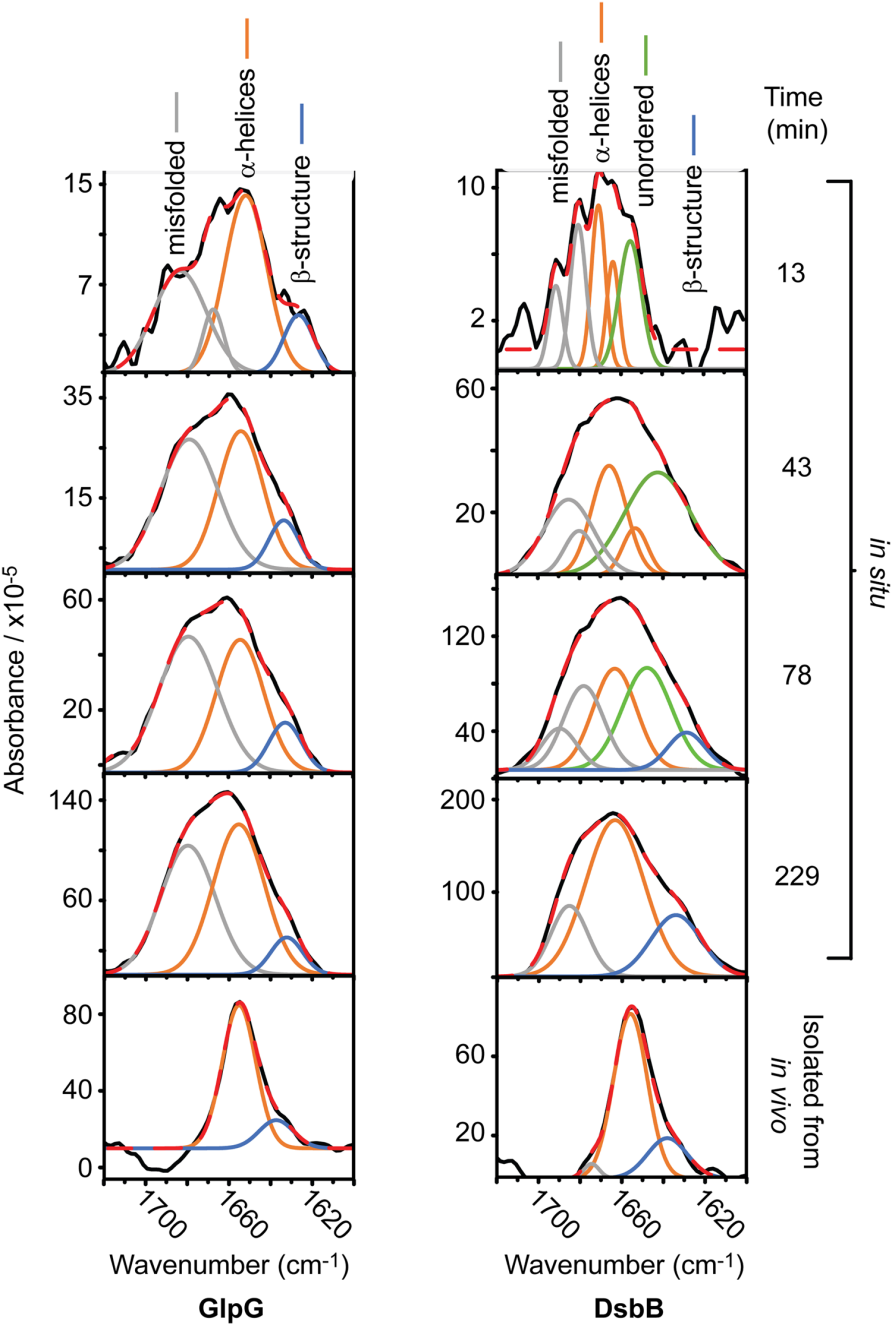
Secondary structure contributions over time during GlpG and DsbB cell-free synthesis. Raw SEIRAS spectra of the amide I band during GlpG (left) and DsbB (right) cell-free synthesis, insertion and folding into DMPC nanodiscs. A change in the proportion of each secondary structure type occurs as folding progresses. The black line is the experimental data and the dashed red line is the cumulative fit of the individual Gaussian contributions. Overall GlpG has lower band intensities in comparison to DsbB; this is likely due to the unfavourable insertion of GlpG into DMPC (Fig. 2). Secondary derivative analysis of the raw FT-IR spectra was used to assist in band position assignment (Fig. S3), with all derived band positions provided in Table S2.

SEIRAS (Fig. 3) shows spontaneous folding of GlpG and DsbB associated with DMPC nanodiscs. The emergence of band positions within the amide I band is due to secondary structure formation, where a band shift is indicative of secondary structure changes (Fig. 4). Protein secondary structure assignment and contribution was assessed from secondary derivative spectra analysis^39^ (Fig. S3 and Table S2). Secondary derivative analysis is used to separate overlapping bands. One of the major advantages of secondary derivative analysis is that it can be performed objectively without arbitrarily choosing deconvolution parameters.

Amide I bands are observed for both GlpG and DsbB at ~13 minutes, during synthesis at 22 °C, which is less than the ~40 mins required to make full length GlpG and DsbB at 24 °C (Fig. S4 and Table S3). Thus, band intensities observed in the first 40 minutes by SEIRAS result from co-translational folding and insertion within the DMPC nanodisc bilayers.

### SEIRAS measurements of GlpG synthesis

The earliest detected stages (13 minutes) of GlpG folding demonstrated the formation of secondary structure populations including α-helices (1651 cm^−1^), β-structure (1623 cm^−1^), and misfolded structure (1684–1668 cm^−1^). After ~40 minutes the misfolded structure contribution reduces with the emergence of α-helices (1661 cm^−1^), whilst the abundance of β-structure regions remains consistent (1631 cm^−1^). The α-helical band absorption maxima for GlpG changes significantly between 10 and 40 minutes - from 1651 to 1661 cm^−1^. This 10 cm^−1^ frequency up-shift is indicative of α-helical bundle formation^32, 40^ implying tertiary structure folding of the polypeptide into its polytopic form. All signatures for α-helices and β-structure regions remained but increased in intensity until approximately five hours, where little change in intensity was observed.

### SEIRAS measurements of DsbB synthesis

The earliest detected stages (13 minutes) of DsbB folding produced mixtures of α-helices (1664 cm^−1^) and unordered structure (1647 cm^−1^), with negligible β-structure (1633 cm^−1^) contribution. Between 10–40 minutes, α-helical content became the most prominent feature (1660 cm^−1^). α-helical peaks remain, and β-structure peaks appear, both growing in intensity over the next five hours. However, a significant presence of unordered structure persists until ~4 hours (<229 minutes) where its contribution eventually becomes drastically reduced. This suggests a late post-translation folding event (as translation is exhausted at ~4 hours using our cell-free systems (Fig. S4)), where significant contributions of unordered structure are folded into α-helices.

### Secondary structure comparison of cell-free, and *in vivo* produced and isolated GlpG and DsbB

The final folded structures of both cell-free and *in vivo* produced GlpG and DsbB show remarkably similar FT-IR spectra, however there are differences in contributions of band positions at >1670 cm^−1^ (Fig. 4, Fig. S3 and Table S2). Band positions >1670 cm^−1^ are regularly attributed to β-turn structures, although here they are most likely signatures of misfolded and/or aggregated membrane protein. The extensive hydrogen bonding in β-structure would produce much stronger band intensities at <1640 cm^−1^ than the less extensive hydrogen bonding within the β-turns (>1670 cm^−1^). We observe much larger intensities at band positions >1670 cm^−1^ than at band positions of <1640 cm^−1^ suggesting these band positions are characteristic of misfolded turn structures. The Gaussian bands are broader in the cell-free spectra than in the *in vivo* produced spectra, this is because the recorded SEIRAS spectra comprise a mixture of folded and misfolded protein. We demonstrate the presence of folded, functional protein in Fig. S2.

The frequency of the final α-helices band for cell-free produced GlpG was almost the same as that of purified GlpG reconstituted into a DMPC bilayer (1661 and 1659 cm^−1^, respectively), with a 6 and 4 cm^−1^ frequency up-shift compared to GlpG in DDM micelles (1655 cm^−1^). DsbB demonstrated similar differences in its α-helical frequencies, with cell-free produced DsbB producing larger frequencies (1663 cm^−1^) than purified DsbB in DDM, and of that reconstituted into a DMPC bilayer (1656 and 1659 cm^−1^, respectively), a 7 and 4 cm^−1^ up-shift.

The observed frequency up-shifts can be explained by different inter-helical coupling among helices, which can significantly influence the amide I absorption of proteins^40^. The frequency up-shift may also be due to the influence of different membrane lipid order, lateral pressure profile and curvature on inter-helical coupling within TMs. A micellar environment offers lower lateral pressure than liposome lipid bilayers, and nanodiscs possess a more ordered, gel-like, and possibly more native bilayer^38^ with higher lateral pressure in comparison to liposomes^41^.

### Chemically denatured GlpG and DsbB aggregate during insertion into lipid membranes

We undertook a study to compare cell-free, co-translationally folded protein with that refolded from chemically-denatured states. For the latter work, fully translated folded protein was denatured and refolded. A range of denaturants and refolding conditions were tested. No significant denaturation was observed for DsbB or GlpG in SDS or urea, and whilst both were denatured by guanidinium hydrochloride (GuHCl), it was not possible to achieve efficient refolding for either protein. Purified, folded and fully translated GlpG and DsbB were denatured in 8 M urea or 6 M GuHCl. Neither GlpG nor DsbB lose any secondary structure, as observed by circular dichroism (CD), upon exposure to 8 M urea. No significant structure loss in SDS/n-dodecyl-β-D-maltoside (DDM) mixed micelles was observed by CD for GlpG^27^ and DsbB^30^. However, GuHCl did denature both GlpG and DsbB (Fig. S5), inducing α-helical losses. Therefore, we attempted to refold both DsbB and GlpG from GuHCl.

The GuHCl chemically denatured state of each protein was diluted ten times into liposomes (final GuHCl concentration 0.6 M) as an attempt at refolding (Fig. S5). Many higher-order oligomers were observed in all lipid compositions (in both the top and bottom fractions of the sucrose flotation), indicating early-stage aggregation regardless of lipid composition and whether insertion was observed. Refolding of GuHCl denatured GlpG into nanodiscs was also investigated using SEIRAS (Fig. S5); predominantly β-structure is observed (associated with aggregated states), with only small amounts of α-helical structure. Although insertion was inefficient due to aggregation, solution phase aggregates will not reside within the 10 nm range of SEIRAS and hence only protein associated within the lipid nanodisc is observed. In both experiments, the inefficient insertion observed may be in part due to perturbations of the bilayer caused by GuHCl, hindering insertion and refolding, demonstrating the advantages of the cell-free approach to studying folding.

SDS solubilised states of GlpG and DsbB (Fig. S6) were also investigated. There was no reduction in secondary structure in SDS for either protein^27, 30^. A small band shift and reduction in intrinsic protein fluorescence was observed for DsbB in SDS, which may represent a small amount of tertiary structure disruption but the change cannot be assigned and could equally reflect aggregation and/or changes in solubilisation of tryptophan in DsbB. Essentially no change in fluorescence was observed for GlpG in SDS, strongly indicating no disruption of structure. When attempting to transfer GlpG and DsbB from a SDS solubilised state into liposomes (final SDS concentration 0.5 mM), most of the protein remained in the bottom fraction of the sucrose gradient, indicating poor membrane insertion and aggregation during this process, despite variations in lipid composition (Fig. S6). This lack of insertion is in line with previous reports for DsbB^28^ which, despite its insignificant structural loss in SDS, did not efficiently transfer into PC lipid vesicles.

The results for chemically denatured proteins are in stark contrast to our cell-free inserted GlpG and DsbB into lipid membranes (Fig. 2), which produced significant amounts of homogenous folded proteins possessing a specific activity comparable to the proteins isolated from *E. coli*.

## Discussion

We have shown that the membrane proteins GlpG and DsbB can insert spontaneously into lipid membranes during co-translational synthesis in the absence of cellular translocase machinery, and can fold efficiently into functional membrane proteins. This provides an efficient experimental system for mechanistic studies of α-helical membrane protein folding, allowing study of co-translational folding during synthesis of the nascent chain, which cannot be mimicked in denaturant folding studies. A significant proportion of the total protein synthesised can spontaneously insert and fold correctly in the membranes; adjustment of the lipid composition led to over 60% of the total protein synthesised spontaneously inserting into the bilayer, and higher yields could be possible with further lipid optimisation.

Our finding that two different α-helical proteins can successfully fold co-translationally into a lipid membrane in the absence of translocase insertion apparatus is consistent with a thermodynamic hypothesis of folding^42^; which states that the three-dimensional native protein structure is that for which the Gibb’s free energy of the system is lowest, and thus is determined by the amino acid sequence in a given environment. Thus, as shown here, it is possible to fold to the final functional state *in vitro*, independent of the native pathway. This adds to previous evidence pointing to membrane proteins being equilibrium structures, including earlier cell-free work^14–22^, and the fact that functional proteins can be recovered in synthetic lipid systems from partly denatured states^8–13, 43^. However, when using partially denatured states to study folding, the remnants of structure present in the starting state of the folding reaction are most likely native core elements that are critical to recover a correct fold. Moreover, in many cases it is not possible to regain the folded state from chemically denatured states, due to misfolding and aggregation.

By altering the composition of the liposomes used in cell-free synthesis we could alter the yield of inserted protein. Lipids influence membrane protein insertion and folding either through specific binding interactions or general bilayer properties including charge, hydrophobic thickness, curvature elastic energy or lateral pressure^44^. Mixing lipids that readily form bilayers with those which do not alters the lateral pressure profile in the bilayer plane, increasing the outward pressure of the lipid chains whilst reducing the pressure in the headgroup/interface region. Such an increase in chain pressure has been found to hinder insertion of helices and proteins across the bilayer and increase the stability of the folded state^11, 13, 43^. In our cell-free experiments, GlpG favours a charged bilayer with high lateral pressure (i.e. 1:1 mol ratio DOPG:DOPE, EPL), while DsbB favours a neutrally charged bilayer with saturated lipid chains and low lateral pressure in the acyl chain region (i.e. DMPC).

SEIRAS measures structure formation during nascent chain folding of DsbB and GlpG into lipid membranes in real-time during on-going protein synthesis, revealing differences in nascent membrane protein folding pathways. At the earliest detected stages DsbB and GlpG associate with the nanodisc as a mixture of secondary structures. Both were predominantly α-helical in structure, suggesting that α-helical structure formation occurred during synthesis, likely within the ribosome tunnel^45^. Only GlpG possessed substantial β-structure at this early stage, whereas little was observed for DsbB. It may be possible to assign this β-structure to the co-translational folding of the partly β-structured N-terminal soluble domain of GlpG, although the current IR spectra do not clearly distinguish β-sheet contribution. At <43 min tertiary structure of GlpG began to form, containing α-helical signatures. In comparison, some unordered structure was present for DsbB at the earliest detected stages of synthesis, and this unordered structure remained until a post-translational folding event occurred at <229 minutes. Interestingly, mixtures of folded and misfolded content were observed even at the earliest folding stages (<13 minutes) suggesting that several folding, and misfolding, pathways are occurring co-translationally for both proteins.

**Figure 5:**
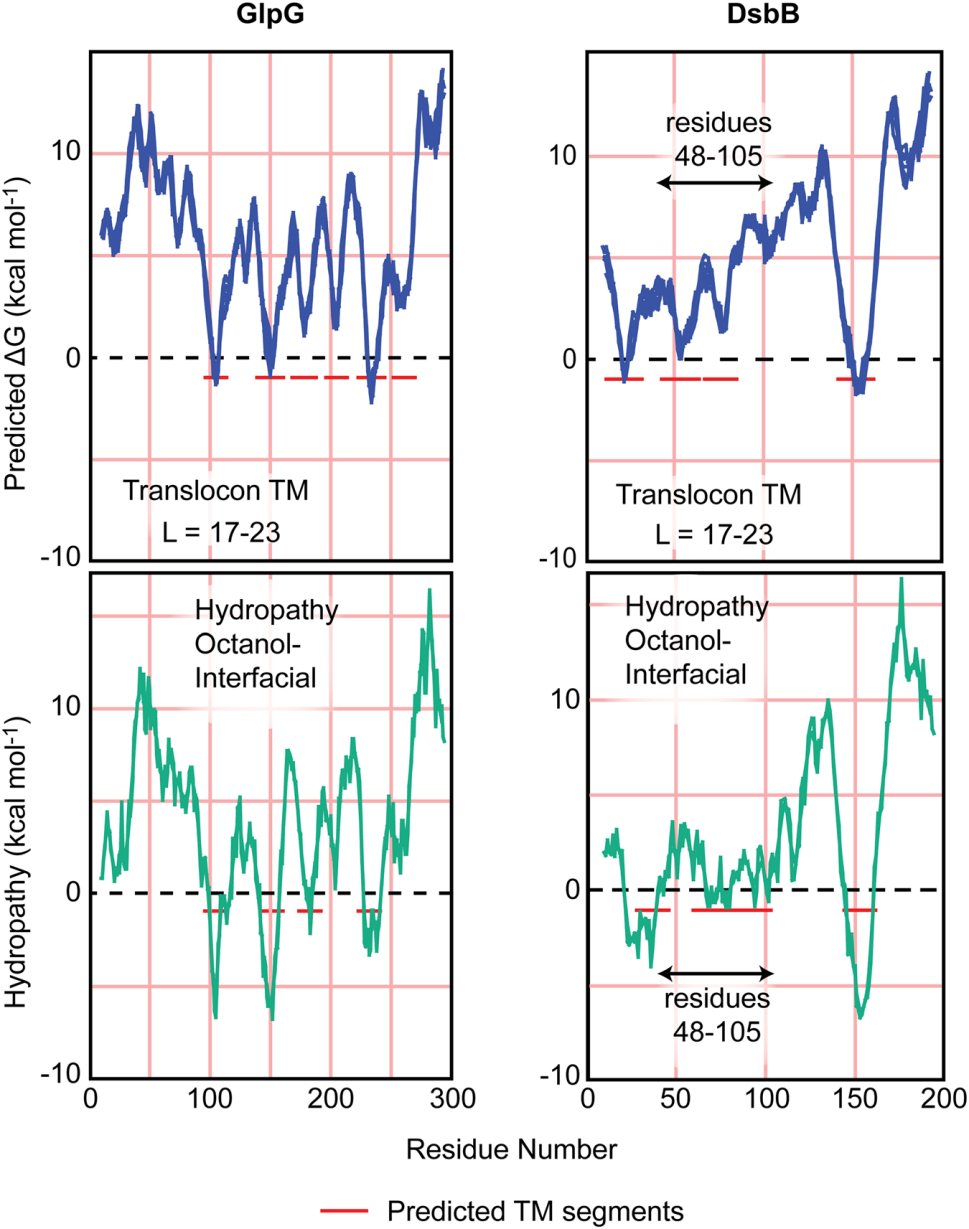
MPEx^46^ analysis of DsbB and GlpG. The translocon TM analysis (top row, blue) analyses the TM transfer free energetics based on translocon-mediated TM helix assembly. The Octanol-Interfacial scale (Oct-IF, bottom row, green) identifies segments that tend to prefer a TM helix conformation relative to an unfolded interfacial location. The translocon TM analysis and Oct-IF analysis for GlpG (left) are remarkably similar. The hydropathy analysis for DsbB (right) reveals substantial differences between the translocon TM and Oct-IF analysis, specifically for the polypeptide region (48–105 residues) corresponding to the second and third TM (TM-2 and TM-3, respectively).

Hydropathy plots were produced for GlpG and DsbB using MPEx (Fig. 5)^46^. MPEx is comprised of two analyses; the translocon TM analysis, which analyses the TM transfer free energetics based on translocon-mediated TM helix assembly, and the Octanol-Interfacial scale (Oct-IF) which identifies segments that tend to prefer a TM helix conformation relative to an unfolded lipid interfacial location. The hydropathy analysis for GlpG revealed that the translocon TM analysis and Oct-IF analyses are remarkably similar and consistent with the first two TM regions (TM-1 and TM-2) of GlpG inserting rapidly into the membrane as the protein emerges from the ribosome. The hydropathy analysis for DsbB revealed substantial differences between the translocon TM and Oct-IF analysis, specifically for the polypeptide region (48–105 residues) corresponding to the second and third TM (TM-2 and TM-3), inferring that the residues corresponding to TM-2 and TM-3 are more favourable as a disordered chain at a PC interface than as a membrane spanning TM helix. This suggests that TM-1 and TM-4 can insert readily into the membrane once translated, but TM-2 and TM-3 insert less favourably, and may only be able to insert once TM-4 is inserted. The late post-translational folding event observed during DsbB folding, where significant populations of unordered structure are lost to emergent α-helical contributions, could be interpreted as TM-2 and TM-3 post-translationally inserting into the membrane. TM-2 of DsbB has been suggested to have topological plasticity^47^ validating that this region of DsbB is a dynamic folding region; translocon unassisted folding and insertion of TM-2 and TM-3 is potentially the cause for our topology results which show that DsbB inserts in an inverse topology.

**Figure 6:**
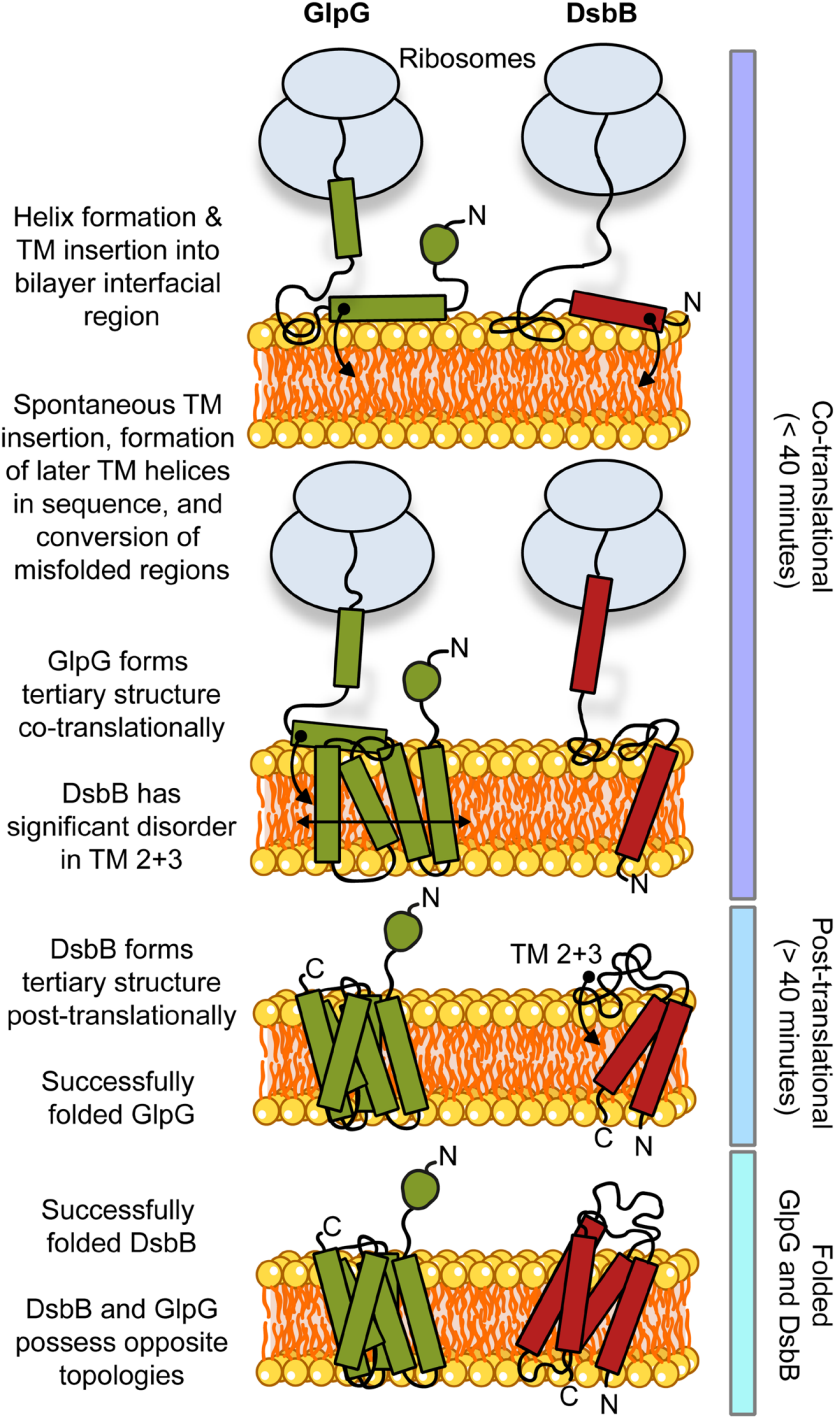
Schematic of postulated co-translational folding pathways for GlpG and DsbB unaided by membrane insertion apparatus. Suggested routes are supported by a combination of time-resolved SEIRAS (Figs 3 and 4 and S3), MPEx analysis (Fig. 5), the crystal structures (Fig. 1), and AMS labelling (Fig. 2). This is only a postulated, simple folding scenario that is consistent with our data. From our protein synthesis rate measurements (Fig. S4), we propose any events <40 minutes observed by SEIRAS are co-translational, whereas events >40 minutes are progressively post-translational. GlpG forms helices and inserts into the bilayer co-translationally, folding to form its polytopic tertiary structure during translation. Insertion is likely driven by the favourable thermodynamics for TM insertion, especially the leading TM-1 and TM-2. In contrast, DsbB folds into its final polytopic form post-translationally, likely related to the less favourable thermodynamics for TM insertion of TM-2 and TM-3 compared to the more favourable TM-1 and TM-4 insertion. The N- and C- termini are labelled N and C respectively. GlpG is shown in green (left) and DsbB in red (right), with the nascent polypeptide chain shown in black and folded helices shown as coloured rectangles; the circular shape at the GlpG N-terminus represents its cytoplasmic domain which possesses some β-structure.

Combining our results, we propose tentative models for DsbB and GlpG insertion based on a combination of the SEIRAS (Figs 3 and 4 and S3), MPEx (Fig. 5), the AMS labelling data (Fig. 2) and the crystal structure (Fig. 1) in Fig. 6. The observed nascent structure formation for GlpG is consistent with the N-terminal cytoplasmic domain folding early (within 10 min) during translation, accompanied by some helix formation and misfolded structures. All six GlpG TM helices are energetically favoured in the membrane interface, as well as inserted across the membrane. Thus, we can postulate that early co-translational folding events involve misfolded and helical structure formation at the membrane interface; the subsequent co-translational events between 10–40 min involve concomitant TM helix formation, membrane insertion, and packing, and include conversion of these interfacial misfolded and helical structures to the GlpG TM helical bundle. Therefore, the stable hydrophobic TM helices of GlpG result in efficient helix formation and packing that seems to occur largely co-translationally, and can correct early misfolded structures at the membrane surface. For DsbB, the lower hydrophobicity and stability of TM-2 and TM-3 results in unordered structure being predicted, most likely at the membrane interface, throughout co-translational folding. Some helical structure also forms during these early events, which presumably reflects the more energetically favoured TM-1 and TM-4 formation. The later, much slower conversion of unordered structure to the TM helical bundle of DsbB seems most likely to occur post-translationally. Thus, in this simple model we propose that TM-2 and TM-3 of DsbB insert after TM-1 and TM-4, consistent with the thermodynamics predicted by MPEx.

Previous *in vitro* folding studies using chemically denatured states (with reduced secondary and tertiary structure) have shown that some proteins spontaneously refold directly into lipid bilayers, with the residual structure present in the denatured state enabling recovery of the native fold. However, there are only a few examples of such successful folding^8–13^, as folding attempts are prone to misfolding and aggregation^10, 48^. Here, co-translational folding studies provide a route to probe folding by avoiding such aggregation as the nascent chain can insert into the bilayer as it is being made. Neither GlpG nor DsbB can be refolded efficiently into lipid membranes from a partially denatured state, with significant aggregation being observed instead. In contrast, insertion occurs efficiently to form the folded state in lipid membranes during cell-free synthesis, as the nascent chain inserts in the absence of any detergents or denaturants. Cell-free methods are therefore an improvement on current methods to study insertion and folding in bilayers, as cell-free folding avoids the use of denaturants altogether.

Our results demonstrate that membrane protein insertion and folding can be driven solely by the thermodynamics dictated by their amino acid sequence – in line with TM helical hydrophobicity - and the lipid bilayer properties. Moreover, we show that in the absence of any translocase insertion machinery membrane proteins can be synthesised by the ribosome and proceed through unique secondary and tertiary structure formation pathways to form a folded and functional membrane protein. We have found that the structural conformations during this process, and the final protein fold and topology, can be explained by the thermodynamic principles described by the physical and ‘biological’ hydrophobicity scales^49–51^. Therefore, in the absence of translocase insertion machinery membrane proteins can successfully insert and fold into a membrane spontaneously, forming a final structure like that formed when *in vivo*. Hence, the essential role of the translocase machinery is likely the targeting and coordinated assembly of membrane proteins to prevent misfolded membrane proteins within the complex and crowded environment of the cell.

## Materials and Methods

### Materials

All standard reagents were purchased from Sigma. The PURExpress® *In Vitro* Protein Synthesis Kit and all molecular biology reagents were purchased from New England Biolabs. Lipids were purchased from Avanti Polar Lipids, and pre-cast SDS-PAGE gels and detergents were purchased from Generon. Other exceptions are included in the text where relevant.

pET28a was used as the vector for GlpG, DsbB, and DsbA, for both cell free expression and *in vivo* overexpression. This was modified with a myc tag and linker between the coding regions and 6x Histidine tag (6xHis). The non-essential Cysteine (C) residues of DsbB, C8 and C49, were mutated to Alanine to aid protein purification^24^ - this mutant is referred to as wild-type DsbB throughout the text. A stop codon was mutated between the DsbA coding region and the myc tag, linker and 6xHis to produce a tag-free expression plasmid.

### Expression and purification of GlpG

GlpG WT (from *E. coli* K12; Genbank ID U18997.1) and its mutants (A8C and S147C) were overexpressed and purified following the same procedure, as follows. GlpG was overexpressed in *E. coli* BL21-AI cells. 100 ml of Luria-Bertani (LB) media containing 30 μg ml^−1^ Kanamycin (LB-KAN) was incubated with shaking overnight at 37 °C with a colony picked from a fresh transformation. This overnight culture was used to inoculate 6 × 1 L of LB-KAN in baffled flasks, which were then grown at 37 °C at 250 rpm. The cells were induced when the OD_600_ reached 0.8 AU, with 0.1% (w/v) arabinose and 1 mM IPTG. When growth became stationary the cells were harvested at 5000 × *g* for 45 mins. The cells were then washed in 200 ml PBS and spun at 6000 × *g* for 15 mins to re-pellet the cells. They were then resuspended in 50 ml PBS and 35 mM β-mercaptoethanol (BME), with a Protease Inhibitor Cocktail Tablet (Roche Applied Science). The cells were then frozen at −20 °C.

To purify GlpG, the cells were defrosted and lysed with a microfluidizer (Constant Systems) at 25,000 psi, and the membranes pelleted by centrifugation at 100,000 × *g*. The membranes were then solubilised in solubilisation buffer (50 mM sodium phosphate (NaPhos) pH 7.0, 300 mM NaCl, 20 mM imidazole, 10 mM BME, 10% (v/v) glycerol, 2% (w/v) *n*-Dodecyl β-*D*-maltoside (DDM) and an EDTA free Protease Inhibitor Cocktail Tablet (Roche Applied Science)), and stirred overnight at 4 °C. The solubilised membranes were then spun at 100,000 × *g* for 30 mins, and the supernatant incubated with 500 µl of pre-equilibrated TALON resin (Clontech) for 1 hr at 4 °C. The resin was then washed with 20 mM imidazole (50 mM NaPhos pH 7.0, 300 mM NaCl, 20 mM imidazole, 10 mM BME, 10% (v/v) glycerol, 0.05% (w/v) DDM), and GlpG was eluted with 250 mM imidazole. The eluted GlpG was then desalted (50 mM NaPhos pH 7.0, 300 mM NaCl, 2 mM BME, 10% (v/v) glycerol, 0.05% (w/v) DDM) with a PD-10 desalting column (Fisher Scientific), aliquoted and frozen at −80 °C. Protein purity was assessed by SDS-PAGE, western blot analysis, and the secondary structure was assessed by circular dichroism (CD).

### Expression and purification of DsbB

DsbB (from *E. coli* K12; Genbank ID AAC74269.3) was overexpressed in *E. coli* BL21-AI cells following the same procedures used for GlpG except that the washed cell pellets were stored at −80 °C before purification.

The cell pellets were resuspended in 20 ml per litre of culture of Buffer A (50 mM NaPhos, pH 8.0, 300 mM NaCl, 0.1 mM PMSF, and 5 mM BME) supplemented with a protease inhibitor cocktail tablet and DNase. The cell suspension was then passed three times through a microfluidizer (Constant Systems) at 25,000 psi. Insoluble material was pelleted at 20,000 × *g* for 25 min at 4 °C. Membranes were pelleted from the supernatant by centrifugation at 100,000 × *g* for 2 hours at 4 °C. The membranes were then resuspended in ice-cold buffer B (50 mM NaPhos, pH 8.0, 300 mM NaCl, 0.1 mM PMSF, 20% (v/v) glycerol, and 5 mM BME) and homogenized using a Potter-Elvehjem Teflon pestle and glass tube.

DsbB was extracted from purified membranes with 1% (w/v) of DDM in buffer B and incubated overnight at 4 °C with gentle agitation. Insoluble material was pelleted at 20,000 × *g* for 25 min at 4 °C. The supernatant was then filtered (using a 0.22 μm filter (Millex)) before loading onto a 1 ml HiTrap (GE Healthcare) equilibrated in buffer C (50 mM NaPhos, pH 8.0, 300 mM NaCl, 20 mM Imidazole, 10% glycerol, and 0.025% (w/v) DDM). The column was washed with 5% buffer D (50 mM NaPhos, pH 8.0, 130 mM NaCl, 500 mM Imidazole, 10% (v/v) glycerol, and 0.025% (w/v) DDM). DsbB was eluted with buffer D and injected directly onto a Superdex 75 10/600 GL size exclusion chromatography (SEC) column (GE Healthcare) equilibrated in buffer E (10 mM HEPES-KOH, pH 7.5, 300 mM NaCl, 10% (v/v) glycerol, and 0.025% (w/v) DDM). Peak fractions eluted from the SEC column containing pure DsbB were pooled, concentrated using a 50 kDa Molecular Weight Cut Off (MWCO) concentrator (Amicon), and spin filtered (using a 0.22 μm filter) before being aliquoted, flash frozen and stored at −80 °C. SDS-PAGE and Western blotting assessed DsbB purification, and the secondary structure was assessed by CD. The concentration of the purified DsbB was determined after reduction of protein-bound quinone with NaBH_4_ using the extinction coefficient of ε_276_ = 46.5 mM^−1^ ^53^.

### Expression and purification of DsbA

DsbA (from *E. coli* K12; Genbank ID CAA56736.1) was overexpressed in *E. coli* BL21(DE3) cells. 50 ml of LB-KAN was incubated overnight at 37 °C with a colony picked from a fresh transformation. This overnight culture was used to inoculate 1 L of LB-KAN in a baffled flask, which was then grown at 37 °C at 250 rpm. The cells were induced when the OD_600_ was 0.8 AU with 0.5 mM IPTG. The cells were then harvested after 1.5 hours at 3,800 × *g* for 40 mins at 4 °C and the supernatant discarded.

To purify the periplasmic DsbA protein the extraction protocols derived by Quan *et al*.^54^ were followed. Briefly, the cell pellet was gently resuspended in 10 ml TSE buffer (200 mM 2-Amino-2-hydroxymethyl-propane-1,3-diol (Tris)-HCl, pH 8.0, 500 mM sucrose, 1 mM EDTA, and 0.1 mM PMSF) supplemented with one protease inhibitor cocktail tablet per litre and incubated on ice for 30 min. The cell suspension was centrifuged at 16,000 × *g* for 30 mins at 4 °C. The supernatant was then centrifuged at 100,000 × *g* for 1 hour at 4 °C to remove outer membrane components.

The supernatant was then filtered and purified using ion-exchange chromatography (IEX), using a strong anion exchanger, equilibrated with buffer F (50 mM Tris-HCl, pH 7.8, 0.1 mM PMSF). DsbA was separated into fractions over a salt concentration of 40–60 mM using buffer G (50 mM Tris-HCl, pH 7.8, 500 mM NaCl, 0.1 mM PMSF). The fractions were pooled, concentrated using a 10 MWCO concentrator (Amicon), and then incubated in 20 mM DTT for 20 min on ice. DsbA then underwent SEC with a Superdex 75 10/600 GL column (GE Healthcare) equilibrated in buffer H (20 mM HEPES-KOH, pH 7.5, 0.5 mM EDTA, and 0.1 mM PMSF). SDS-PAGE electrophoresis and western blotting, with an anti-DsbA antibody (Abcam), assessed DsbA purification. Pure DsbA was then supplemented with glycerol (10% final glycerol concentration), spin filtered (using a 0.22 μm filter), aliquoted, flash frozen, and stored at −80 °C. The thiol content was measured with Ellman’s reagent and found to be >95% reduced.

### Liposome preparation

DOPC, DOPE, DOPG and *E. coli* polar extract (EPL) were dissolved in cyclohexane at 50 mg ml^−1^ and mixed at the desired ratios. DMPC was dissolved in chloroform at 50 mg ml^−1^. Some of the lipid compositions contained rhodamine-DOPE (1,2-dioleoyl-*sn*-glycero-3-phosphoethanolamine-N-(lissamine rhodamine B sulfonyl)), at a final concentration of 0.02%. All the lipid mixtures were dried down with a dry stream of nitrogen and frozen in liquid nitrogen, and the remaining solvent removed under vacuum to make lipid films. All lipid films were stored at −20 °C. To make liposomes, the lipid films were suspended at 10 mg ml^−1^ in 40 mM HEPES-KOH pH 7.6, with vortexing and stirring. The lipids were then extruded through a 100 nm or 400 nm filter (Avanti Polar Lipids), with at least 31 pushes, to create liposomes. These were then used immediately in a cell-free reaction.

### Reconstitution of GlpG and DsbB

1% OG was added to the extruded liposomes, and then protein was added at a lipid:protein ratio of 10,000:1 for GlpG and 7,500:1 for DsbB. The detergent was removed using Biobeads (BioRad) with three changes of beads, each incubated for one hour. A Markwell-Lowry assay^55^ was used to determine the protein concentration in the liposomes. The reconstituted protein was then ready for further experiments.

### Cell-free synthesis and insertion of GlpG and DsbB

The PURExpress® *In Vitro* Protein Synthesis Kit (New England Biolabs) was used for all experiments. Reactions were set up following the manufacturer’s instructions, supplemented with previously prepared liposomes (at a final lipid concentration of 3–5 mg.ml^−1^), and a protease inhibitor solution used to make the reaction up to the required volume. Reactions were typically 12.5, 25 or 50 µl, with 50 ng.μl^−1^ plasmid DNA. The reaction was then incubated at 30 °C for 2–3 hours. For reactions containing [^35^S]Methionine (PerkinElmer), 0.04–0.1 mCi.ml^−1^ was used in the cell free reaction. For reactions containing L-[^14^C(U)]-Leucine (PerkinElmer) 6 μCi.ml^−1^ was used. To measure the rate of elongation, 2 µl of the cell free reaction was removed at each time point, and pipetted onto 0.45 μm MF-Membrane (Millipore). The protein made was quantified according to the PURExpress® *In Vitro* Protein Synthesis Kit manual and Wuu and Swartz^56^, with a 1600 TR Tri-Carb® Liquid Scintillation Counter (Packard) and using the Ultima Gold MV scintillation cocktail (PerkinElmer).

#### GlpG membrane insertion

All urea and sucrose solutions were prepared in 40 mM HEPES-KOH pH 7.6. Following the cell-free reaction, 6 M urea was added to a volume of 200 µl, and the liposomes pelleted at 350,000 × *g* for 30–60 mins. The liposomes were resuspended in 50 µl 60% sucrose, and 100 µl of 30% sucrose was layered on top, followed by 50 µl of 40 mM HEPES-KOH pH 7.6. The sucrose gradients were then spun at 200,000 × *g*, to float the liposomes to the 30% sucrose:buffer interface. The sucrose layers were then pipetted off carefully, either to be loaded directly onto an SDS-PAGE gel, or to be used for further analysis. The liposomes float to the buffer:30% sucrose interface, an indication that they are intact and have not been adversely affected by the addition of urea.

For detection of protein using western blotting, the sucrose gradient fractions were loaded onto a 12% SDS-PAGE gel and transferred onto a nitrocellulose membrane. An anti-myc antibody was used to detect cell free expressed protein. For reactions containing [^35^S]Methionine, 2 µl of each sucrose layer and 2 µl of the total reaction was pipetted onto 0.45 μm MF-Membranes. The amount of GlpG in each sucrose layer was then quantified by liquid scintillation counting as described above.

#### DsbB membrane insertion

Membrane insertion was detected using a similar method to that described by Wuu and Swartz ^56^. Briefly, the cell free reaction was dialysed against 200 volumes of dialysis buffer (20 mM HEPES-KOH, pH 7.6, 100 mM KCl, and 1 mM dithiothreitol (DTT)), three times, for >3 hours in a 12–14 kDa MWCO dialysis membrane (Medicell Membranes).

The cell-free reaction was then diluted in buffer X (20 mM HEPES-KOH, pH 7.4, 100 mM KCl, 1 mM DTT, 50% sucrose, and 6.7 M urea) to yield a final concentration of 45% sucrose and 6 M urea. Sucrose gradients were prepared by layering on buffer Y (20 mM HEPES-KOH, pH 7.4, 100 mM KCl, 1 mM DTT, 25% sucrose, and 6 M urea) and then buffer Z (20 mM HEPES-KOH, pH 7.4, 100 mM KCl, 1 mM DTT, and 6 M urea) on top at a volume ratio of 0.5:1:0.5 (buffer X:Y:Z). When performing topology and activity assays DTT and urea was omitted and the sucrose concentrations were adjusted to 45% and 65% to match the previous urea:sucrose solution densities (omission of urea and DTT made negligible differences to the amount of DsbB present in the top fractions). The sucrose gradients were then spun at 237,000 × *g* for 1–2 hours at 23 °C using a TLS-55 rotor. After centrifugation, fractions were carefully collected using a Hamilton syringe from the top of the gradient and the distribution of radiolabeled protein was determined by liquid scintillation counting of the collected fractions, as described above.

For cell-free expression in the presence of DMPC nanodiscs, the nanodiscs were added to the cell free reaction to a final concentration of 80 µM. After incubation at 30 °C for 2–3 hours the reaction was pelleted in a microcentrifuge at 12,000 × *g* for 20 mins to remove the insoluble fraction, and the supernatant analysed by western blotting and assayed for function.

### Denatured membrane protein membrane insertion

Over-expressed and purified GlpG and DsbB were incubated in either 6 M guanidine hydrochloride (GuHCl) or in mixed micelles of 5 mM DDM and 5 mM SDS (a SDS mole fraction (χSDS) of 0.5). 3 μM of each protein was used during GuHCl denaturation, in 20 mM HEPES, pH 7.6 and unfolded at 25 °C for 5 mins (see Fig. S5). 5 μM of each protein was used for SDS incubation, in 20 mM HEPES, pH 7.6 and incubated at 25 °C for 2 hr. Both conditions were then diluted ten-fold into liposomes of each lipid composition (at a final lipid concentration 3 mg.ml^−1^, and a final GuHCl concentration of 0.6 M or SDS concentration of 0.5 mM) in buffer: 33 mM HEPES-KOH, pH 7.6, 100 mM potassium glutamate, 13 mM magnesium acetate, 2 mM spermidine, and 1 mM DTT (this buffer composition was chosen to emulate the PURE system buffer^14^). Previous work has shown that SDS promotes insertion^11, 12, 57^, and that liposomes are intact in 0.5 mM SDS^58^ and 0.6 M GuHCl^59^. The reaction was then incubated for 2 hr at 30 °C as performed during cell-free synthesis of GlpG and DsbB. The liposomes were resuspended to 100 µl in 45% sucrose/6 M urea (20 mM HEPES-KOH, pH 7.6), then 100 µl of 25% sucrose/6 M urea (20 mM HEPES-KOH, pH 7.6) was layered onto the bottom fraction, and finally 50 µl of 20 mM HEPES-KOH, pH 7.6 was layered on top. The sucrose gradients were then spun at 237,000 × *g*, to float the liposomes to the 25% sucrose:buffer interface. Top and bottom 125 μl fractions were then pipetted off carefully, to be analysed by western blotting with an anti-myc tag antibody.

### Fluorescence spectroscopy

Fluorescence emission spectra of over-expressed and purified GlpG and DsbB were taken before and after SDS denaturation, and after refolding into liposomes. GlpG was excited at 280 nm, and DsbB at 295 nm^27, 30^, and the fluorescence emission of each measured from 310–400 nm (Fluoromax-4, Horiba Scientific). Fluorescence measurements of cell-free reactions containing rhodamine-DOPE used an excitation wavelength of 560 nm (emission band at 590 nm).

### Assessing the activity of GlpG

The protease activity of reconstituted and cell free inserted GlpG was assayed using BODIPY-casein as provided by the EnzChek® Protease Assay Kit (green fluorescence, Life Technologies). Although BODIPY-casein is not a native substrate of GlpG, this assay has been used previously to assess GlpG activity^23^. Reconstituted GlpG in 1:1 DOPG:DOPE liposomes or GlpG from the top and bottom sucrose gradient fractions of a cell-free insertion experiment were made up to 120 µl using the reaction buffer supplied with the EnzChek® kit, and were incubated with a final concentration of 5 µg.ml^−1^ substrate. The reactions were incubated overnight at 4 °C, and the fluorescence emission measured at 23 °C between 500–600 nm following excitation at 480 nm, with the substrate emission band at around 516 nm (Fluoromax-4, Horiba Scientific).

### Assessing the activity of DsbB

The activity of cell free produced and recombinantly expressed DsbB was assessed using an assay previously described by Bader *et al*.^25^. Purified reduced DsbA was further incubated with 1 mM DTT for 20 min on ice before being buffer exchanged into reaction buffer (50 mM NaPhos, pH 5.8, 300 mM NaCl, and 0.5 mM EDTA) using a Micro Bio-Spin^TM^ column (Bio-Rad) to remove the DTT. Reduced DsbA (2 μM) was then incubated with saturating amounts of ubiquinone Q1 (Sigma Aldrich, 25 μM) and the reaction started by addition of catalytic amounts of cell free DsbB sucrose floated fractions or purified *in vivo* produced DsbB. The decrease in ubiquinone Q1 absorbance at 275 nm was monitored at 23 °C using a Cary 300 Bio UV-Vis spectrophotometer (Varian). For DDM solubilised DsbB samples the reaction buffer also contained 0.025% DDM.

### Surface-Enhanced InfraRed Absorption Spectroscopy (SEIRAS)

Measurements of GlpG and DsbB by SEIRAS were performed as described previously^32^, using the MembraneMax™ Protein Expression Kit (Life Technologies) for cell free expression in the presence of ~10 nm DMPC nanodiscs (supplied with MembraneMax™), at 22 ± 1 °C and pH 7.4. Plasmid DNA at a concentration of 50 ng.μl^−1^ was used for both GlpG and DsbB. GlpG nanodiscs were eluted from the SEIRAS surface by addition of 75 mM NaPhos, pH 7.8, 125 mM EDTA, and assayed to assess the function of inserted GlpG. A Protease Inhibitor Cocktail Tablet (Roche Applied Science) was added during the functional assay to prevent any protease activity of the cell free reaction contents (GlpG is unaffected by common protease inhibitors)^36^.

Protein secondary structure information was obtained from the SEIRAS spectra using established protocols described by Yang *et al*.^39^. Essential FTIR® software was used to buffer subtract, baseline, and find the second-derivative of the FT-IR spectra, as well as perform minimal smoothing of the second-derivative spectra. The peak frequency of the second derivative is identical to the original peak frequency. The half-width of the second derivative (γ^II^) is related to the half-width of the original line (γ) by γ/γ^II^ = 2.7. A least-squares fitting procedure within OriginPro software was used to fit the secondary structure components to a Gaussian shaped curve.

### Circular Dichroism (CD)

Purified GlpG and DsbB (buffer exchanged into 100 mM NaPhos, pH 7.0, 0.025% DDM using a Micro Bio-spin column (BioRad)) were measured by CD in a rectangular 0.1 mm cell. The concentrations of GlpG and DsbB were 0.7 and 0.5 mg ml^−1^, respectively. CD scans with 6 M GuHCl and 8 M urea were measured in a 0.2 mm cell, at a protein concentration of 0.3 and 0.2 mg ml^−1^ for GlpG and DsbB, respectively. The data was collected at 25 °C, from 260–190 nm at 1 nm intervals with an averaging time of 1 s. The buffer background was subtracted from the spectra using CDtool^60^, and the data analysed using the SMP180 dataset (optimized for 190–240 nm) on DichroWeb^61, 62^.

### AMS labelling

4-Acetamido-4′-Maleimidylstilbene-2,2′-Disulfonic Acid (AMS) (ThermoFisher) was used to measure exposure of Cys residues on the outside of the liposomes^63^.

For GlpG, 5 µl of the top sucrose gradient fraction was incubated overnight at 4 °C, both with and without 10 mM AMS, and with and without 1% DDM. Labelling was quenched by addition of SDS loading buffer, containing BME, and the samples were run on a 12% SDS-PAGE gel. GlpG was visualized by western blot analysis with an anti-myc tag antibody.

For DsbB, 5–10 µl of the sucrose floated fractions, prepared as described above but with reducing agent omitted from all buffers, were incubated with 1 mM of membrane impermeable 3,3′,3′′-phosphanetriyltripropanoic acid (TCEP), and with or without AMS (stock concentration of 100 mM AMS in 20 mM HEPES-KOH, pH 7.6, 100 mM KCl) for 30 min at room temperature. Labelling was then quenched by addition of SDS loading buffer, containing BME, and the samples were run on a 12% SDS-PAGE gel. DsbB was visualized by western blot using a polyvinylidene fluoride (PVDF) membrane.

## Electronic supplementary material

Supplementary Information
