# Supplementary material for: Structure formation during translocon-unassisted co-translational membrane protein folding
Source: Sci Rep. 2017 Aug 14;7:8021. doi: 10.1038/s41598-017-08522-9 (PMC5556060; doi:10.1038/s41598-017-08522-9)
Supplement: Supplementary file 1 — Supplementary Information [file 41598_2017_8522_MOESM1_ESM.pdf]

Structure formation during translocon-unassisted co-translational membrane protein folding

Nicola J. Harris[1†], Eamonn Reading[1†], Kenichi Ataka[2], Lucjan Grzegorzewski[2], Kalypso Charalambous[1], Xia Liu[3], Ramona Schlesinger[2], Joachim Heberle[2], Paula J. Booth[1*]

1. Department of Chemistry, Britannia House, 7 Trinity Street, King's College London, London, UK
2. Department of Physics, Freie Universität Berlin, Arnimallee 14, 14195 Berlin-Dahlem, Germany
3. School of Biochemistry, Medical Sciences, University Walk, University of Bristol, Bristol, UK

[†]N.J.H. and E.R. contributed equally to this work.

[*]To whom correspondence may be addressed. Email: paula.booth@kcl.ac.uk

Author contributions: N.J.H., E.R., K.C., K.A., R.S., J.H., and P.J.B. designed the research. X.L. contributed essential reagents. N.J.H. and E.R. performed all experiments except for SEIRAS experiments. K.A., L.G., and R.S. performed SEIRAS experiments, and K.A., L.G., and E.R. analysed SEIRAS data. N.J.H., E.R., and P.J.B. wrote the manuscript with contributions from all authors.

Supplementary Discussion

DsbB topology

DsbB posits a more complex system for topology interrogation due to the inability to produce a functional cysteine-less construct, as there are four essential cysteine residues present within its periplasmic loops. The DsbB construct used throughout this study was the double mutant C8A/C49A, where two non-essential cysteine residues were knocked out (as performed for previous biochemical and structural studies[1]). Advantageously, this left only the cysteines present within the two disulphide bonds on the periplasmic loops available, upon reduction, for AMS modification. It has been shown that DsbB can be modified in this way, resulting in a mass shift observable with SDS-PAGE and western blot analysis[2]. It is posited that by reducing the system with membrane impermeable TCEP, only cysteines exposed on the surface (outside) of the liposomes will be reduced and therefore available for AMS modification. Liposome solubilisation (with DDM) produced a mobility shift upon increasing AMS incubation, suggesting that most the cell-free produced DsbB inserts with its periplasmic loops exposed on the outside of DMPC liposomes – an opposite topology to that found *in vivo* and for GlpG. This topology is likely the result of the highly unfavourable cost of translocating the hydrophilic domains of DsbB across the hydrophobic membrane.

Supplementary Figures and Tables

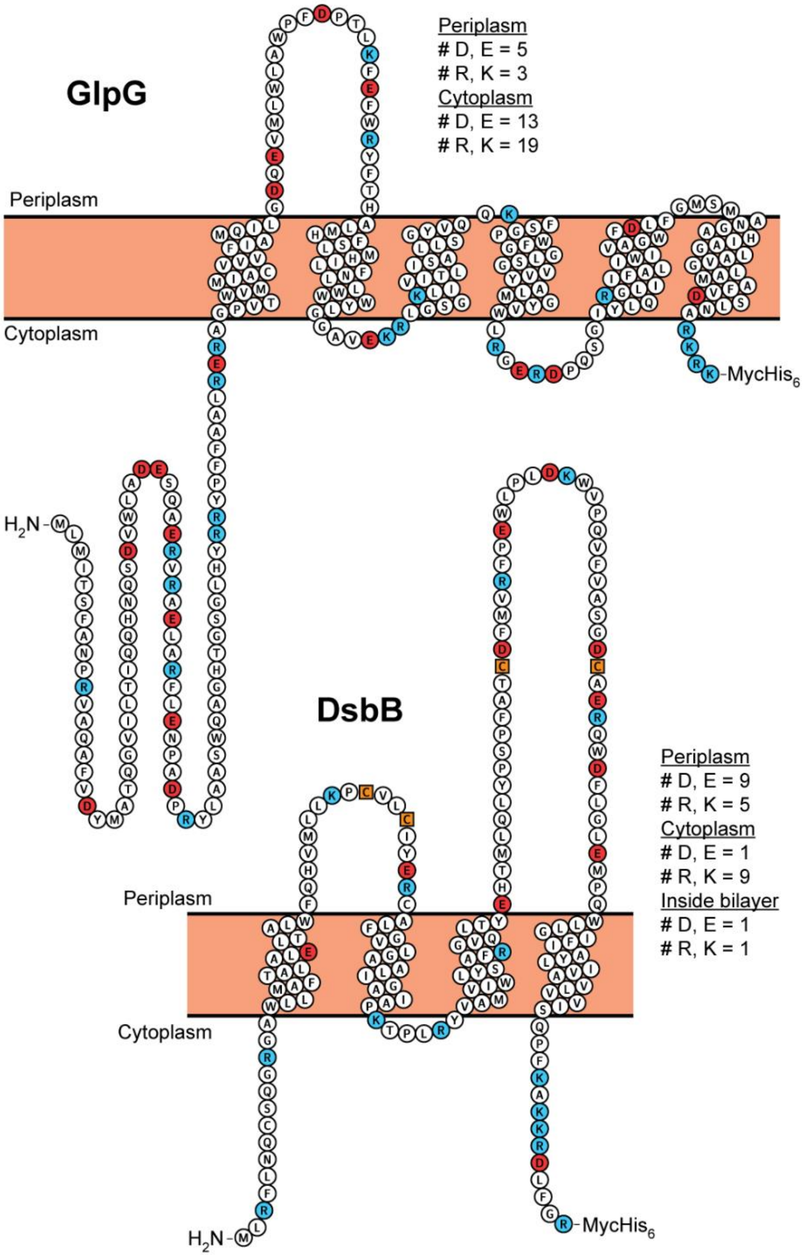

Supplementary Figure 1. Charge distribution in GlpG and DsbB, with residues (K and R) shown in blue, and residues (D and E) shown in red, and disulphide forming Cys residues in orange. Our cell-free experiments were performed at pH 7.6, therefore the residues K and R are likely positively charged, and D and E negatively charged during our experiments.

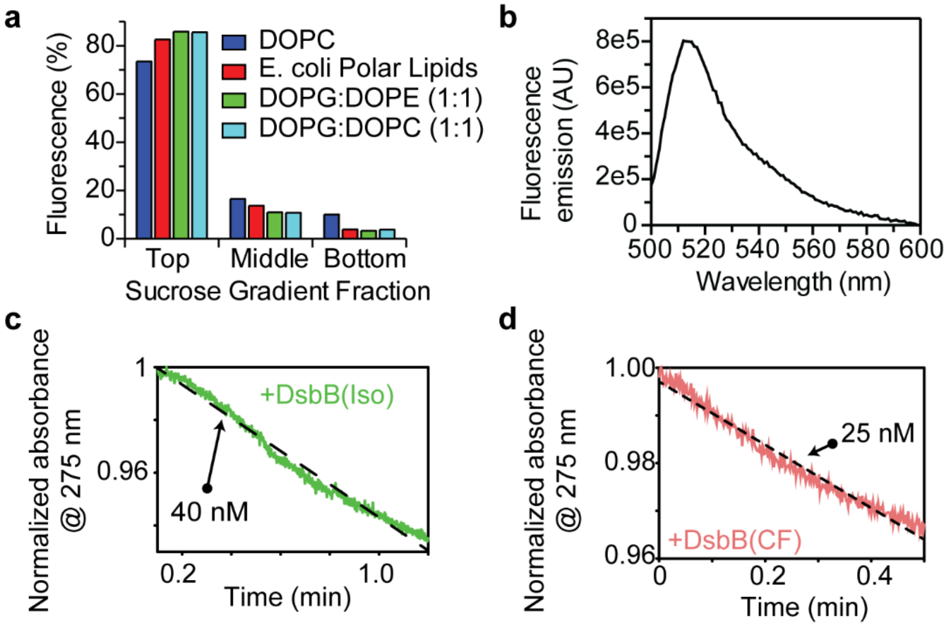

Supplementary Figure 2. Liposome flotation analysis, and GlpG and DsbB function. (**a**) Liposomes float the same on a sucrose gradient regardless of the lipid composition. Rhodamine-DOPE was added at a concentration of 0.02 % (w/v) to each lipid composition, and the liposomes were floated on a sucrose gradient following cell-free insertion. The rhodamine fluorescence in each layer was measured to ascertain the number of liposomes present (excitation 560 nm, emission measured from 570 – 650 nm, emission band at 590 nm). (**b**) GlpG produced by cell-free synthesis using MembraneMax™ Protein Expression Kit at 22 °C, pH 7.4 into DMPC nanodiscs on a SERIAS surface is functional. The histidine tagged nanodiscs attached to the SEIRAS surface were eluted with 125 mM EDTA and assayed for GlpG protease activity. BODIPY casein protease digestion by GlpG results in a fluorescence emission band at 520 nm after excitation at 480 nm. The lower fluorescence intensity compared to GlpG expressed in cell-free reactions in bulk (**Fig. 2**), as opposed to on a surface, is due to the smaller amount of GlpG present in the assay. (**c** and **d**) DsbB redox functional assay with DsbA and ubiquinone; reduction of ubiquinone results in a strong decrease in absorbance at 275 nm. *In vivo* expressed and isolated DsbB in DDM

micelles (**c**, Iso, green line) or cell-free produced DsbB in DMPC nanodiscs (**d**, CF, pink line). Least-squared fitted linear fits (dashed black lines) were used to calculate the initial rate of quinone reduction.

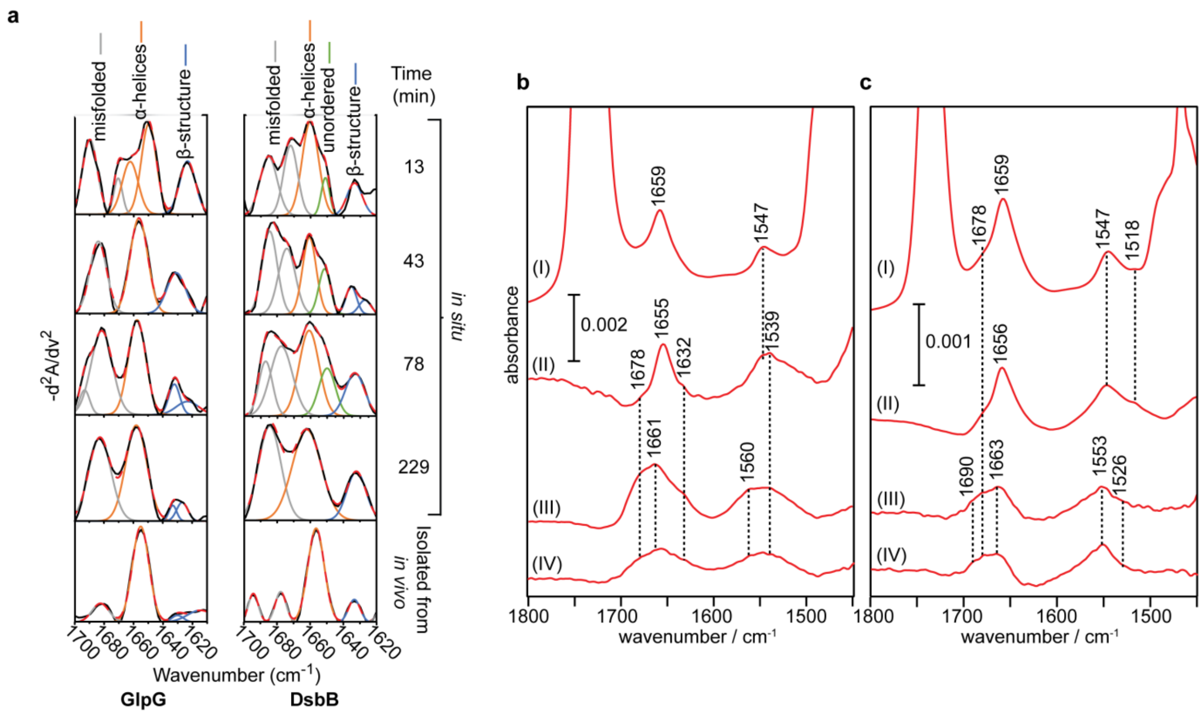

Supplementary Figure 3. SEIRAS analysis of GlpG and DsbB during cell-free synthesis and when isolated from *in vivo*. (**a**) Second derivative of the amide I peaks in **Fig. 4**. All band positions can be found in **Table S2**. A band for β-structure can be fitted for the DsbB spectra at 13 min and 43 min, which cannot be fitted in the raw IR spectra (**Fig. 4**). Therefore, there are only significant β-structure contributions after 43 min. Spectral comparison of purified and cell-free produced DsbB (**b**), and GlpG (**c**). The spectra labelled (I) are ATR-FTIR spectra of isolated membrane protein reconstituted into DMPC liposomes. The spectra labelled (II) are SEIRA spectra of isolated membrane protein in DDM micelles, adsorbed onto an Ni-NTA modified gold surface via a C-terminal His-tag. The spectra labelled (III) and (IV) are SEIRA spectra of cell-free expressed samples, but observed in different experiments to confirm the reproducibility of the measurement.

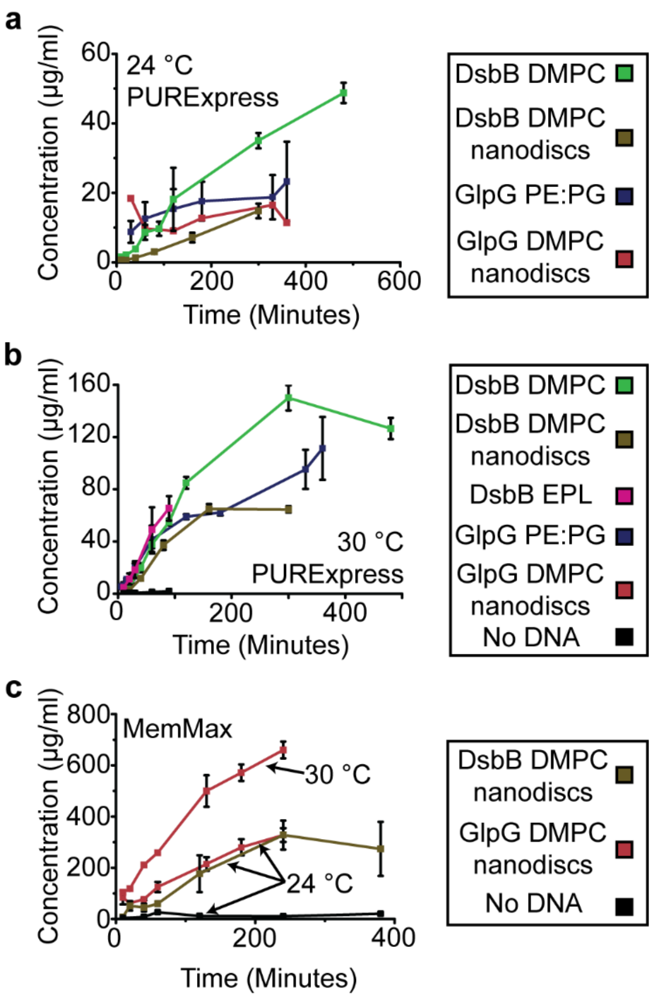

Supplementary Figure 4. Protein synthesis rates of DsbB and GlpG. (**a**) Cell-free expression of GlpG and DsbB measured at 24 °C using PURExpress. (**b**) Cell-free expression of GlpG and DsbB measured at 30 °C using PURExpress. (**c**) Cell-free expression of GlpG and DsbB measured at 30 °C and 24 °C using MembraneMax. The rate of protein production is much slower at 24 °C than at 30 °C. The commercialized *E. coli* crude extract MembraneMax kit has a faster synthesis rate than PURExpress at the same temperature. It has been shown previously that the PURExpress system possesses significant cold sensitivity in comparison to commercialized *E. coli* crude extracts; it was stated that the PURExpress cold-sensitivity could be due to its lack of certain cellular factors whose *in vivo* functions are to maintain efficient translation after cold shock[3]. Interestingly, PURExpress at 30 °C and MembraneMax at 24 °C produced similar rates of protein synthesis (**Table S2**). Zhang *et al*[4] found *in vivo* translation rates at 24 °C to be 0.4 aa/sec, which decreased by a factor of five or two

when compared to the rates at 37 °C (2.3 (aa)/sec) and 30 °C (0.7 (aa)/sec), respectively. In our SEIRAS experiments we therefore approximate a translation rate of ~0.13 (aa)/sec for both DsbB and GlpG – this around 3 times lower than that expected *in vivo,* which is expected as *in vitro* systems elongation rates are typically slower than *in vivo* rates (sometimes even being an order of magnitude slower)[3].

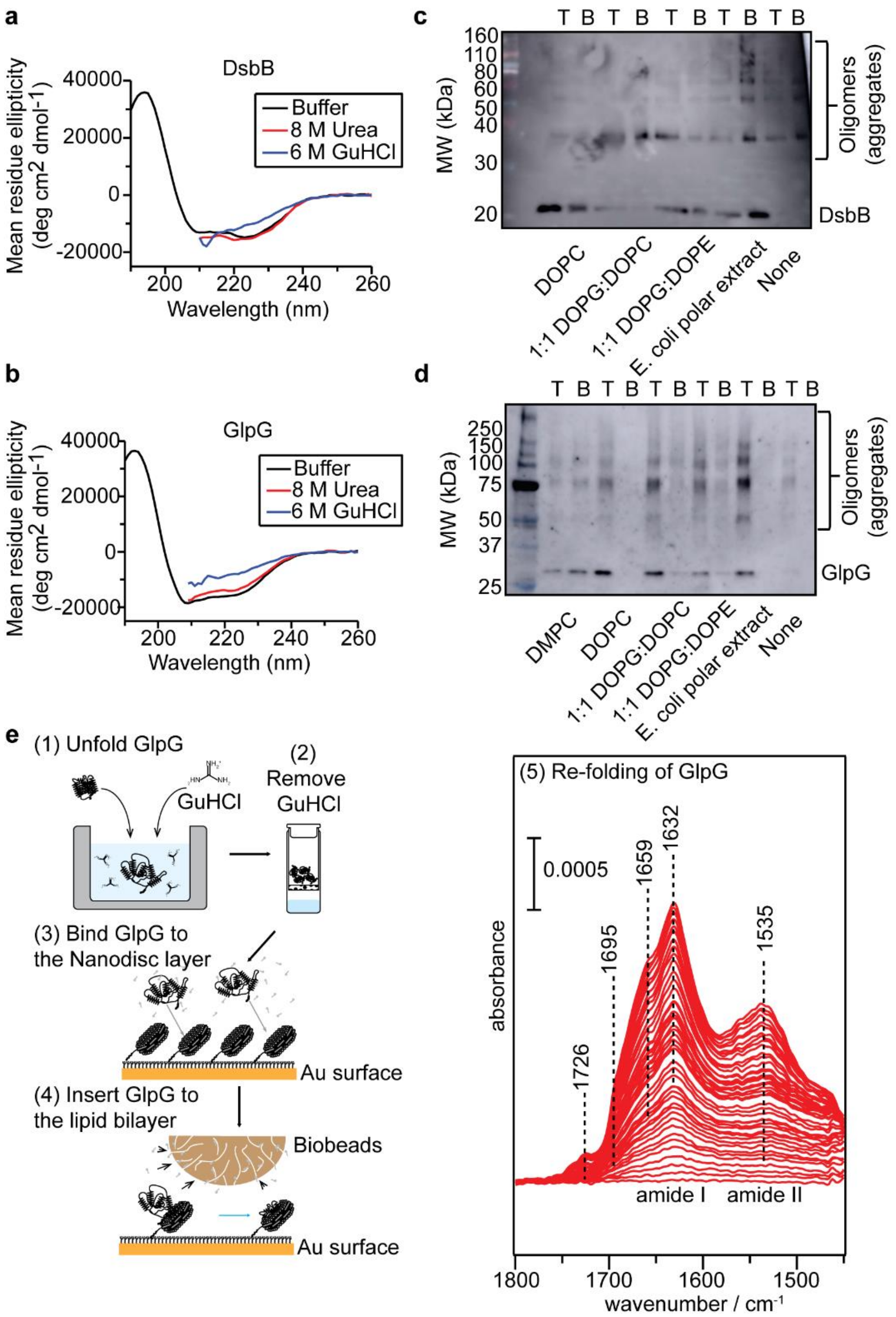

Supplementary Figure 5. Unfolding and refolding of denatured GlpG and DsbB into liposomes. Far-UV CD spectra of DsbB (**a**) and GlpG (**b**) isolated from *in vivo*, and with the addition of 8 M urea (red) or 6 M GuHCl (blue). The spectra reflect the predominant α-helical structure. Only in 6 M GuHCl do the proteins lose α-helical structure (as judged by a reduction in the 222 nm band), GlpG loses around of third of its structure, and DsbB around a quarter of its structure. Western blot analysis of GuHCl denatured DsbB (**c**) and GlpG (**d**) refolded into liposomes of varying composition by dilution of

denaturant. There are many higher-order oligomers present in all samples, in both the top floated and protein inserted (T), and bottom and non-inserted protein (B) fractions of the sucrose gradient, indicating early-stage aggregation regardless of lipid composition and whether insertion was observed. These results indicate that inserting partially denatured, fully translated GlpG and DsbB into liposomes is a highly aggregation-prone process. The absence of protein in the bottom lanes when no liposomes are present can be attributed to large aggregates either not entering the gel, or not transferring onto the membrane. (**e**) Monitoring the refolding of GlpG, isolated from *in vivo*, into DMPC nanodiscs after GuHCl denaturation using SEIRAS. With time a broad absorbance peak emerges with the dominating band position being at 1632 cm^{-1}; this is attributed to GlpG forming a large degree of β-structure. There were also contributions from band positions at 1659 cm^{-1} and 1695 cm^{-1}, which are likely from α-helical and misfolded structure, respectively.

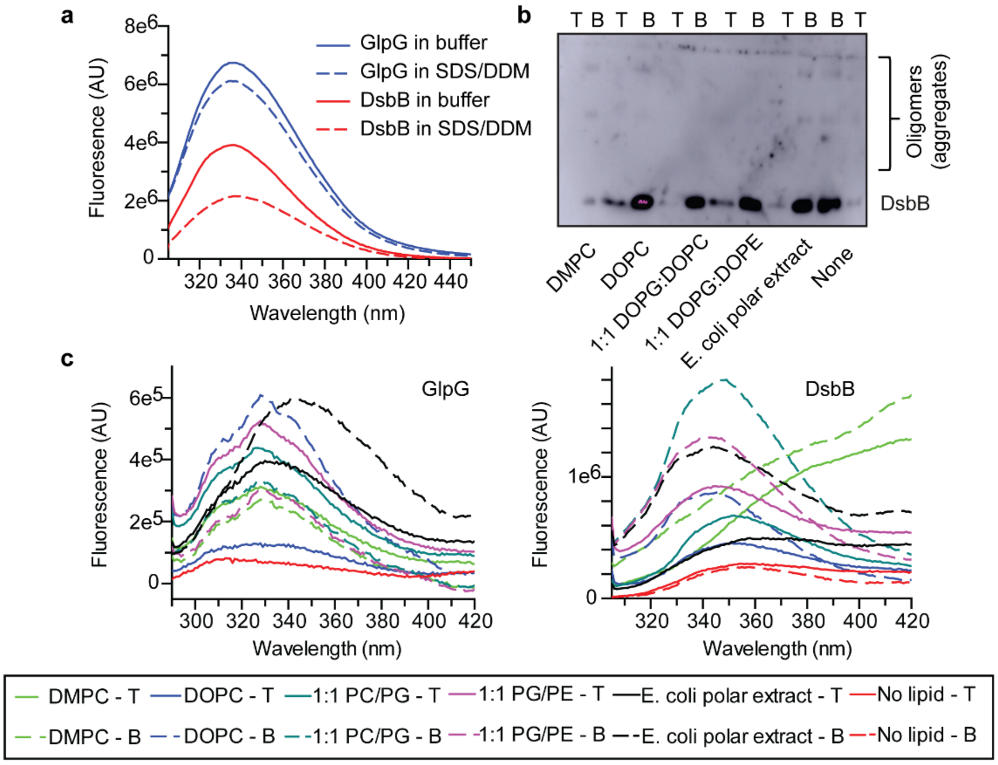

Supplementary Figure 6. SDS/DDM mixed micelle solubilisation and transfer into liposomes. (**a**) We observed a fluorescence maximum of 336 nm for folded DsbB, and 338 nm for SDS/DDM incubated DsbB, as well as decrease in fluorescence intensity. We observed slight blue shifted spectra for GlpG; 336 nm for folded GlpG and 335 nm for SDS/DDM incubated GlpG - thus, only a 1 nm band shift occurred in the fluorescence upon SDS addition, with only a small reduction in fluorescence intensity. These band shifts and intensity changes are most likely due to a difference in buffers and protein solvation. (**b**) Western blot analysis of DsbB within SDS/DDM mixed micelles transferred by dilution into liposomes of varying lipid composition. Most the protein remains in the bottom fraction indicating poor membrane insertion during this process. When GlpG was disrupted within SDS/DDM mixed micelles and transferred to liposomes of varying lipid composition no protein was observed on the nitrocellulose western membrane – indicative of aggregated protein. This contrasts with the results obtained when using our cell-free system (see **Fig. 2**). (**c**) Fluorescence analysis of the top inserted (T) and bottom non-inserted (B) fractions of SDS/DDM mixed micelle disrupted DsbB and GlpG refolded into liposomes of varying lipid composition. Most the protein remains in the bottom

fractions. Fluorescence spectra of the top fraction, which would contain inserted protein, are dissimilar to that of folded GlpG, with considerable lower intensity. The results corroborate with the Western blot analysis.

Supplementary Table 1. Summary table of DsbB functional assays.

Isolated or cell-free DsbB	**Lipid/detergent system**	**Rate (nmol quinone min^{-1})**	**R^2**	**nM DsbB**	**Specific activity (quinone per nmol of DsbB min^{-1})**
Isolated	DDM detergent	5135 ± 24.5	0.99	40	128 ± 0.6
Isolated	DMPC proteoliposomes	4996 ± 65.3	0.95	40	125.0 ± 1.6
Cell-free	DMPC proteoliposomes	1184 ± 16.3	0.99	5	237 ± 3.3
Cell-free	DMPC proteoliposomes	2050 ± 5.0	1.00	14	146 ± 0.4
Cell-free	DMPC nanodiscs	5396 ± 49.0	0.98	25	216 ± 2.0

Supplementary Table 2. Summary table of SEIRAS spectra wavenumbers, fits, and secondary structure assignment for both raw data and secondary (2nd) derivative spectra.

Exp.	Time (min) **Raw data score (R^2)**, ***2nd derivative (R^2)***	Raw data wavenumber (cm^{-1})	2nd derivative wavenumber (cm^{-1})	Assigned secondary structure
		**DsbB**		
In-situ	13 (**0.91**, ***0.91***)	1694	-	β-turns/'misfolded'
		1680	1685	β-turns/'misfolded'
		1672	1672	β-turns/'misfolded'
		1664	1660	α-helix
		1647	1651	Unordered
		1633	1633	β-structure
	43 (**1.00**, ***0.99***)	1685	1684	β-turns/'misfolded'
		1678	1674	β-turns/'misfolded'
		1660	1660	α-helix
		1643	1651	Unordered
		1633	1635	β-structure
		-	1627	β-structure
	78 (**1.00**, ***1.00***)	1690	1687	β-turns/'misfolded'
		1678	1678	β-turns/'misfolded'
		1663	1661	α-helix
		1643	1650	Unordered
		1632	1633	β-structure
	229 (**1.00**, ***0.99***)	1678	1685	β-turns/'misfolded'
		1663	1662	α-helix
		1632	1632	β-structure
	498 (**1.00**, ***0.98***)	1684	1684	β-turns/'misfolded'
		1663	1662	α-helix
		1636	1632	β-structure
DDM	N/A (**0.98**, ***0.99***)	-	1694	β-turns
		1675	1678	β-turns
		1656	1656	α-helix
		1638	1633	β-structure
		**GlpG**		
In-situ	13 (**0.98**, ***0.97***)	1684	1690	β-turns/'misfolded'
		1668	1671	β-turns/'misfolded'
		-	1662	α-helix
		1651	1650	α-helix
		1627	1623	β-structure
	43 (**0.99**, ***0.97***)	1680	1684	β-turns/'misfolded'
		1661	1656	α-helix
		1634	1631	β-structure
	78 (**0.99**, ***0.99***)	-	1693	β-turns/'misfolded'
		1676	1682	β-turns/'misfolded'
		1661	1657	α-helix
		1633	1632	β-structure
		-	1623	β-structure
	229 (**1.00**, ***0.99***)	1676	1683	β-turns/'misfolded'
		1661	1658	α-helix
		1633	1633	β-structure
		-	1627	β-structure
	498 (**0.98**, ***0.99***)	1676	1684	β-turns/'misfolded'

		1661	1659	α-helix
		1633	1634	β-structure
		-	1627	β-structure
DDM	N/A (**0.97**, ***0.99***)	-	1682	β-turns
		1655	1655	α-helix
		1637	1630	β-structure
		-	1615	β-structure

Supplementary Table 3. Summary table of approximate translation rates calculated for the *in vitro* cell-free systems. The initial rate (between 0-120 minutes) of protein synthesis (protein concentration) was fitted to a linear relationship ($f(t) = mt + c$) using a least-squared fitting program. The activity (m) for the ribosomes *in vitro* was calculated as μg ml^{-1} protein/min. Accounting for the reaction mixture volume and ribosome concentration (ribosome concentrations for the PURExpress kit are approximately 2000 nM ± 20 %, and in typical commercialized *E. coli* crude extract kits (e.g. 5 PRIME RTS kit) is approximately 1600 nM[5]) the specific activity may be expressed as pmol protein/pmol ribosome/min. Since this calculated value represents the entire process of translation, a comparison requires the bold assumption that elongation is the rate-limiting step in this process. Correspondingly, under this model, the initial elongation rate (amino acids (aa)/sec) can be calculated for our proteins[6] (**Table S2**). These calculations are approximations to rationalize the earliest events observed during SEIRAS measurements and should not be used or interpreted as explicit rates of DsbB and GlpG synthesis.

Protein	***In vitro* system**	**Temperature (°C)**	**Ribosome activity (ug ml^{-1}/min)**	**R^2**	**Specific activity (pmol protein/pmol ribosome/min)**	**(aa)/sec**
DsbB	PURExpress	24	N/A	N/A	N/A	N/A
		30	0.70	0.99	0.015	0.05
	MembraneMax	24	1.41	0.92	0.038	0.13
GlpG	PURExpress	24	N/A	N/A	N/A	N/A
		30	0.50	0.98	0.007	0.04
	MembraneMax	24	1.39	0.98	0.025	0.13
		30	4.04	0.97	0.073	0.37

Supplementary References

1. Inaba, K. et al. Crystal structure of the DsbB-DsbA complex reveals a mechanism of disulfide bond generation. *Cell* **127**, 789-801 (2006).
2. Regeimbal, J. & Bardwell, J.C.A. DsbB Catalyzes Disulfide Bond Formation de Novo. *Journal of Biological Chemistry* **277**, 32706-32713 (2002).
3. Hillebrecht, J.R. & Chong, S. A comparative study of protein synthesis in in vitro systems: from the prokaryotic reconstituted to the eukaryotic extract-based. *BMC Biotechnology* **8**, 1-9 (2008).
4. Zhang, G., Hubalewska, M. & Ignatova, Z. Transient ribosomal attenuation coordinates protein synthesis and co-translational folding. *Nat Struct Mol Biol* **16**, 274-280 (2009).
5. Li, J., Gu, L., Aach, J. & Church, G.M. Improved Cell-Free RNA and Protein Synthesis System. *PLoS ONE* **9**, e106232 (2014).
6. Capece, M.C., Kornberg, G.L., Petrov, A. & Puglisi, J.D. A simple real-time assay for in vitro translation. *RNA* **21**, 296-305 (2015).
